# Humans and Deep Networks Largely Agree on Which Kinds of Variation Make Object Recognition Harder

**DOI:** 10.3389/fncom.2016.00092

**Published:** 2016-08-31

**Authors:** Saeed R. Kheradpisheh, Masoud Ghodrati, Mohammad Ganjtabesh, Timothée Masquelier

**Affiliations:** ^1^Department of Computer Science, School of Mathematics, Statistics, and Computer Science, University of TehranTehran, Iran; ^2^CerCo UMR 5549, Centre National de la Recherche Scientifique – Université de ToulouseToulouse, France; ^3^Department of Physiology, Monash UniversityClayton, VIC, Australia; ^4^Neuroscience Program, Biomedicine Discovery Institute, Monash UniversityClayton, VIC, Australia; ^5^Institut National de la Santé et de la Recherche Médicale, U968Paris, France; ^6^Sorbonne Universités, UPMC Univ Paris 06, UMR-S 968, Institut de la VisionParis, France; ^7^Centre National de la Recherche Scientifique, UMR-7210Paris, France

**Keywords:** rapid invariant object recognition, ventral stream models, feed-forward vision, deep networks, 2D and 3D object variations

## Abstract

View-invariant object recognition is a challenging problem that has attracted much attention among the psychology, neuroscience, and computer vision communities. Humans are notoriously good at it, even if some variations are presumably more difficult to handle than others (e.g., 3D rotations). Humans are thought to solve the problem through hierarchical processing along the ventral stream, which progressively extracts more and more invariant visual features. This feed-forward architecture has inspired a new generation of bio-inspired computer vision systems called deep convolutional neural networks (DCNN), which are currently the best models for object recognition in natural images. Here, for the first time, we systematically compared human feed-forward vision and DCNNs at view-invariant object recognition task using the same set of images and controlling the kinds of transformation (position, scale, rotation in plane, and rotation in depth) as well as their magnitude, which we call “variation level.” We used four object categories: car, ship, motorcycle, and animal. In total, 89 human subjects participated in 10 experiments in which they had to discriminate between two or four categories after rapid presentation with backward masking. We also tested two recent DCNNs (proposed respectively by Hinton's group and Zisserman's group) on the same tasks. We found that humans and DCNNs largely agreed on the relative difficulties of each kind of variation: rotation in depth is by far the hardest transformation to handle, followed by scale, then rotation in plane, and finally position (much easier). This suggests that DCNNs would be reasonable models of human feed-forward vision. In addition, our results show that the variation levels in rotation in depth and scale strongly modulate both humans' and DCNNs' recognition performances. We thus argue that these variations should be controlled in the image datasets used in vision research.

## 1. Introduction

As our viewpoint relative to an object changes, the retinal representation of the object tremendously varies across different dimensions. Yet our perception of objects is largely stable. How humans and monkeys can achieve this remarkable performance has been a major focus of research in visual neuroscience (DiCarlo et al., 2012). Neural recordings showed that some variations are treated by early visual cortices, e.g., through phase- and contrast-invariant properties of neurons as well as increasing receptive field sizes along the visual hierarchy (Hubel and Wiesel, 1962, 1968; Finn et al., 2007). Position and scale invariance also exist in the responses of neurons in area V4 (Rust and DiCarlo, 2010), but these invariances considerably increase as visual information propagates to neurons in inferior temporal (IT) cortex (Brincat and Connor, 2004; Hung et al., 2005; Zoccolan et al., 2005, 2007; Rust and DiCarlo, 2010), where responses are highly consistent when an identical object varies across different dimensions (Cadieu et al., 2013, 2014; Yamins et al., 2013; Murty and Arun, 2015). In addition, IT cortex is the only area in the ventral stream which encodes three-dimensional transformations through view specific (Logothetis et al., 1994, 1995) and view invariant (Perrett et al., 1991; Booth and Rolls, 1998) responses.

Inspired by these findings, several early computational models (Fukushima, 1980; LeCun and Bengio, 1998; Riesenhuber and Poggio, 1999; Masquelier and Thorpe, 2007; Serre et al., 2007; Lee et al., 2009) were proposed. These models mimic feed-forward processing in the ventral visual stream as it is believed that the first feed-forward flow of information, ~ 150 ms post-stimulus onset, is usually sufficient for object recognition (Thorpe et al., 1996; Hung et al., 2005; Liu et al., 2009; Anselmi et al., 2013). However, the performance of these models in object recognition was significantly poor comparing to that of humans in the presence of large variations (Pinto et al., 2008, 2011; Ghodrati et al., 2014).

The second generation of these feed-forward models are called deep convolutional neural networks (DCNNs). DCNNs involve many layers (say 8 and above) and millions of free parameters, usually tuned through extensive supervised learning. These networks have achieved outstanding accuracy on object and scene categorization on highly challenging image databases (Krizhevsky et al., 2012; Zhou et al., 2014; LeCun et al., 2015). Moreover, it has been shown that DCNNs can tolerate a high degree of variations in object images and even achieve close-to-human performance (Cadieu et al., 2014; Khaligh-Razavi and Kriegeskorte, 2014; Kheradpisheh et al., 2016b). However, despite extensive research, it is still unclear how different types of variations in object images are treated by DCNNs. These networks are position-invariant by design (thanks to weight sharing), but other sorts of invariances must be acquired through training, and the resulting invariances have not been systematically quantified.

In humans, early behavioral studies (Bricolo and Bülthoff, 1993; Dill and Edelman, 1997) showed that we can robustly recognize objects despite considerable changes in scale, position, and illumination; however, the accuracy drops if the objects are rotated in depth. Yet these studies used simple stimuli (respectively paperclips and combinations of geons). It remains largely unclear how different kinds of variation on more realistic object images, individually or combined with each other, affect the performance of humans, and if they affect the performance of DCNNs similarly.

Here, we address these questions through a set of behavioral and computational experiments in human subjects and DCNNs to test their ability in categorizing object images that were transformed across different dimensions. We generated naturalistic object images of four categories: car, ship, motorcycle, and animal. Each object carefully varied across either one dimension or a combination of dimensions, among scale, position, in-depth and in-plane rotations. All 2D images were rendered from 3D object models. The effects of variations across single dimension and compound dimensions on recognition performance of humans and two powerful DCNNs (Krizhevsky et al., 2012; Simonyan and Zisserman, 2014) were compared in a systematic way, using the same set of images.

Our results indicate that human subjects can tolerate a high degree of variation with remarkably high accuracy and very short response time. The accuracy and reaction time were, however, significantly dependent on the type of object variation, with rotation in-depth as the most difficult dimension. Interestingly, the results of deep neural networks were highly correlated with those of humans as they could mimic human behavior when facing variations across different dimensions. This suggests that humans have difficulty to handle those variations that are also computationally more complicated to overcome. More specifically, variations in some dimensions, such as in-depth rotation and scale, that change the amount or the content of input visual information, make the object recognition more difficult for both humans and deep networks.

## 2. Materials and methods

### 2.1. Image generation

We generated object images of four different categories: car, motorcycle, ship, and animal. Object images varied across four dimensions: scale, position (horizontal and vertical), in-plane and in-depth rotations. Depending on the type of experiment, the number of dimensions that the objects varied across were determined (see following sections). All two-dimensional object images were rendered from three-dimensional models. There were on average 16 different three-dimensional example models per object category (car: 16, ship: 18, motorcycle: 16, and animal: 15). The three-dimensional object models are constructed by O'Reilly et al. (2013) and are publicly available.

The image generation procedure is similar to our previous work (Ghodrati et al., 2014). To generate a two-dimensional object image, first, a set of random values were sampled from uniform distributions. Each value determined the degree of variation across one dimension (e.g., size). These values were then simultaneously applied to a three-dimensional object model. Finally, a two-dimensional image was generated by taking a snapshot from the transformed three-dimensional model. Object images were generated with four levels of difficulty by carefully controlling the amplitude of variations across four levels, from no variation (level 0, where changes in all dimensions were very small: Δ_*Sc*_ = ±1%, Δ_*Po*_ = ±1%, $\Delta_{RD}=\pm 1^{{}^{\circ}}$, and $\Delta_{RP}=\pm 1^{{}^{\circ}}$; each subscript refers to one dimension: Sc: Scale, Po: Position, RD: in-depth rotation, RP: in-plane rotation; and Δ is the amplitude of variations) to high variation (level 3: Δ_*Sc*_ = ±60%, Δ_*Po*_ = ±60%, $\Delta_{RP}=\pm 90^{{}^{\circ}}$, and $\Delta_{RD}=\pm 90^{{}^{\circ}}$). To control the degree of variation in each level, we limited the range of random sampling to a specific upper and lower bounds. Note that the maximum range of variations in scale and position dimensions (Δ_*Sc*_ = ±60% and Δ_*Po*_ = ±60%) are chosen in a way that the whole object completely fits in the image frame.

**Figure 1:**
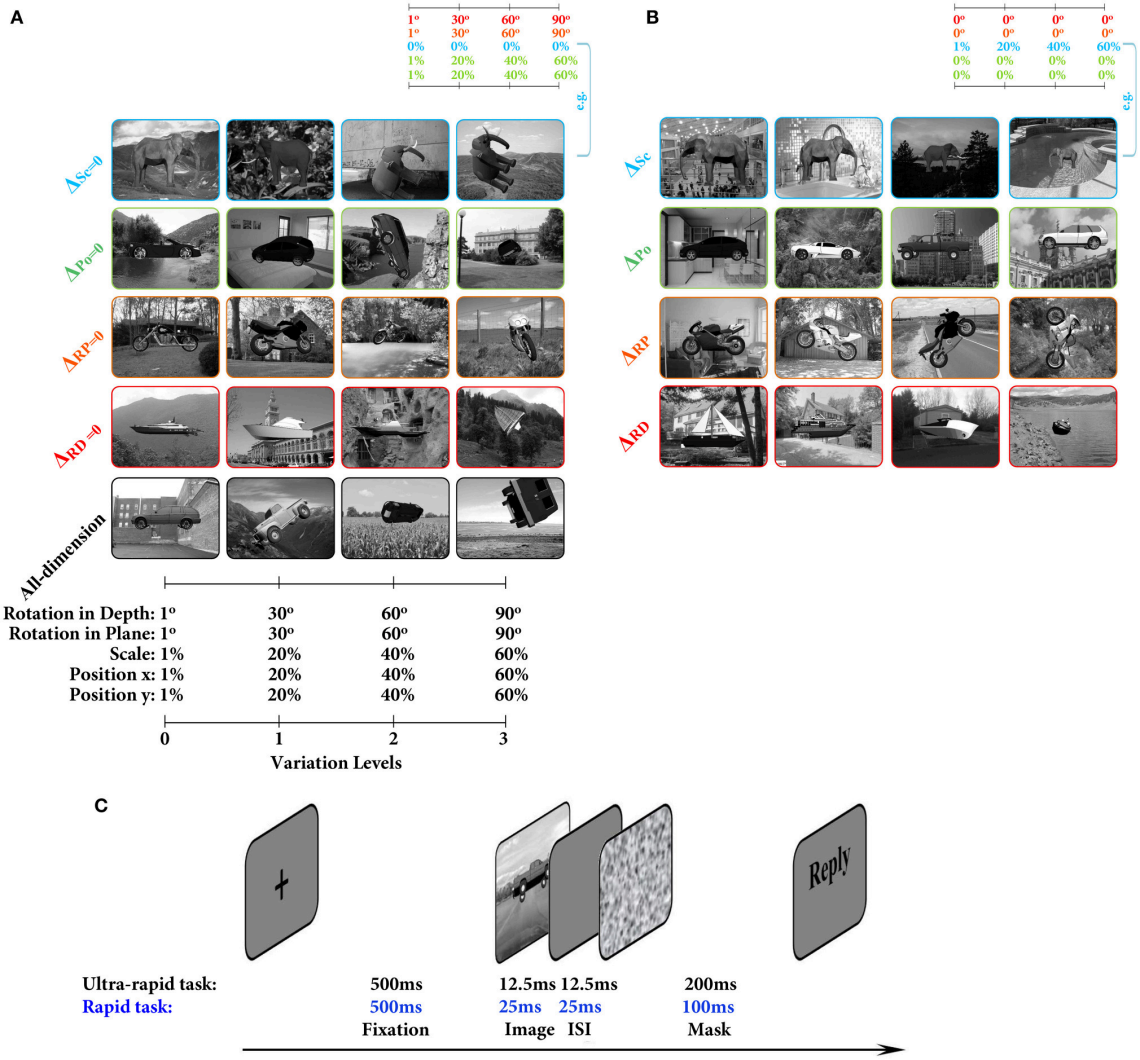
**Image databases and the paradigm of the psychophysical experiment. (A)** Sample object images from all- and three-dimension databases for different categories and variation levels. Each column indicates a variation level and each row refers to a database. First four rows are images from three-dimension database: 1st row: Δ_*Sc*_ = 0; 2nd row: Δ_*Po*_ = 0; 3rd row: Δ_*RD*_ = 0; and 4th row: Δ_*RP*_ = 0. The last row shows sample images from all-dimension database. The range of variations across different levels is depicted below the images. Colored frames refer to the type of database (this color code is the same throughout the paper). **(B)** Sample images from the one-dimension databases. Each row corresponds to one type of database: 1st row: Δ_*Sc*_, Scale-only; 2nd row: Δ_*Po*_, Position-only; 3rd row: Δ_*RP*_, In-plane-only; 4th row: Δ_*RD*_, In-depth-only. The range of variation in each level is the same as **(A)**. **(C)** Psychophysical experiment for rapid and ultra-rapid object categorization (see Materials and Methods).

Several sample images and the range of variations across four levels are shown in Figure 1. The size of two-dimensional images was 400 × 300 pixels (width × height). All images were initially generated on uniform gray background. Moreover, identical object images on natural backgrounds were generated for some experiments. This was done by superimposing object images on randomly selected natural backgrounds from a large pool. Our natural image database contained 3907 images which consisted of a wide variety of indoor, outdoor, man-made, and natural scenes.

#### 2.1.1. Different image databases

To test humans and DCNNs in invariant object recognition tasks, we generated three different image databases:

- **All-dimension:** In this database, objects varied across all dimensions, as described earlier (i.e., scale, position, in-plane, and in-depth rotations). Object images were generated across four levels in terms of variation amplitude (Level 0–3, Figure 1A).
- **Three-dimension:** The image generation procedure in this database was similar to all-dimension database, but object images varied across a combination of three dimensions only, while the forth dimension was fixed. For example, objects' size were fixed across all variation levels while other dimensions varied (i.e., position, in-plane, and in-depth rotations). This provided us with four databases (see Figure 1A): (1) Δ_*Sc*_ = 0: objects' size were fixed to a reference size across variation levels; (2) Δ_*Po*_ = 0: objects' position were fixed at the center of the image; (3) Δ_*RP*_ = 0: objects were not rotated in plane; (4) Δ_*RD*_ = 0: objects were not rotated in depth (three-dimensional transformation) across variation levels.
- **One-dimension:** Object images in this database varied across only one dimension (e.g., size), meaning that the variations across other dimensions were fixed to reference values. Thus, we generated four databases (see Figure 1B): (1) Δ_*Sc*_: only the scale of objects varied; (2) Δ_*Po*_: only the position of objects across vertical and horizontal axes varied; (3) Δ_*RD*_: objects were only rotated in depth; (4) Δ_*RP*_: objects were only rotated in plane.

### 2.2. Human psychophysical experiments

We evaluated the performance of human subjects in invariant object recognition through different experiments and using different image databases. In total, the data of 89 subjects (aged between 23 and 31, mean = 22, 39 female and 50 male) were recorded. Subjects had normal or corrected-to-normal vision. In most experiments, data of 16–20 sessions were recorded (two experiments were run for 5 sessions); therefore, some subjects completed all experiments and others only participated in some experiments. All subjects voluntarily participated to the experiments and gave their written consent prior to participation. Our research adhered to the tenets of the Declaration of Helsinki and all experimental procedures were approved by the ethic committee of the University of Tehran.

Images were presented on a 17″ CRT monitor (LG T710BH CRT; refresh rate 80 Hz, resolution 1280 × 1024 pixels) connected to a PC equipped with an NVIDIA GeForce GTX 650 graphic card. We used MATLAB (www.mathworks.com) with psychophysics toolbox (Brainard, 1997; Pelli, 1997) (http://psychtoolbox.org) to present images. Subjects had a viewing distance of 60 cm and each image covered ~10 × 11 degrees of visual angle.

Details of each experiment are explained in the following sections. Generally, we used rapid image presentation paradigm with mask to only account for the feed-forward processing in the ventral visual pathway (see Figure 1C). Each trial was started with a black fixation cross, presented at the center of the screen for 500 ms, followed by a randomly selected image from the database that was presented for either one or two frames depending on the experiment type (see the description for each experiment). Afterwards, a gray blank screen was presented as inter-stimulus interval (ISI). Finally, a 1/*f* noise mask image was presented. The timing of the image presentation, ISI, and mask depended on the experiment type (see the following sections). Subjects' task was to categorize the presented object images. Subjects were asked to keep their focus around the fixation cross. They were instructed to respond as fast and accurate as they could by pressing a key on computer keyboard (each key was labeled with a category name). The next trial was started after the key press and there was a random time delay before the start of the next trial. We recorded subjects' reaction times (reported in Supplementary Information) and accuracies. Each experiment was divided into a number of blocks and subjects could rest between blocks.

In a training phase prior to the main experiment, subjects performed some practice trials. During the training, for each image, subjects received a feedback indicating if their decision was correct or not. Images in the practice trials were not presented in the main experiments. Every subject performed 40 training trials. It was sufficient for them to understand the task and reach reasonable precision.

#### 2.2.1. Rapid invariant object categorization

In these experiments, subjects categorized rapidly presented images from four object categories (see Figure 1). Each trial started with a fixation cross presented at the center of the screen for 500 ms. An image was then randomly selected from the pool and was presented for 25 ms (2 frames of 80 Hz monitor) followed by a gray blank screen for 25 ms (ISI). Immediately after the blank screen, a 1/*f* noise mask image was presented for 100 ms. Subjects were asked to rapidly and accurately press one of the four keys, labeled on keyboard, to indicate which object category was presented. The next trial started after a key press with a random time delay (2 ± 0.5 s). This experiment was performed in two types that are explained as following:

- **Using all-dimension database:** We used all-dimension database where object images varied across all dimensions (i.e., scale, position, in-plane and in-depth rotations). In each session, subjects were presented with 320 images: 4 categories × 4 levels × 20 images per category. Images were presented into two blocks of 160 images. For each background condition (i.e., uniform and natural backgrounds), we recorded the data of 16 different sessions.
- **Using three-dimension databases:** In this experiment, we used the three-dimension databases. Using these databases, we could measure how excluding variations across one dimension can affect human performance in invariant object recognition: if the fixed dimension is more difficult than the others, subjects would be able to categorize the object more accurately and within shorter time than in the case where the fixed dimension is much easier. We presented subjects with 960 images: 4 categories × 4 levels × 4 conditions (Δ_*Sc*_ = 0, Δ_*Po*_ = 0, Δ_*RP*_ = 0, and Δ_*RD*_ = 0) × 15 images per category. Images were presented in four blocks of 240 images. Note that we inter-mixed images of all conditions in each session; therefore, subjects were unaware of the type of variations. We recorded the data of 20 sessions for the case of objects on natural backgrounds and 17 sessions for objects on uniform background.

To have more accurate reaction times, we also performed two-category (car vs. animal) rapid invariant object categorization tasks with similar experimental settings. The details of these two-category experiments and their results are presented in Supplementary Information.

#### 2.2.2. Ultra-rapid invariant object categorization

To assess whether the experimental design (presentation time and variation conditions) could affect our results and interpretations, we run two ultra-rapid invariant object categorization tasks, using three-dimension and one-dimension databases. In each trial, we presented a fixation cross for 500 ms. Then, an image was randomly selected from the pool and presented to the subject for 12.5 ms (1 frame at 80 Hz monitor). The image was then followed by a blank screen for 12.5 ms. Finally, a noise mask was presented for 200 ms. Subjects had to accurately and rapidly press one of the four keys, labeled on the keyboard, to declare their responses. The next trial started after a key press with a random time delay (2 ± 0.5 s). As mentioned above, this experiment was performed in two types that are explained as following:

- **Using three-dimension databases:** We recorded the data of five sessions. Object images were selected from three-dimension database with natural backgrounds. Images are identical to those of three-dimension rapid presentation experiment described in previous section. But, here, images were presented for 12.5 ms followed by 12.5 ms blank and then 200 ms noise mask.
- **Using one-dimension databases:** In this experiment, we used one-dimension databases with natural backgrounds to evaluate the effect of variations across individual dimensions on human performance. Subjects were presented with 960 images: 4 categories × 4 levels × 4 conditions (Δ_*Sc*_, Δ_*Po*_, Δ_*RP*_, Δ_*RD*_) × 15 images per category. The experiment was divided into four blocks of 240 images. We collected the data of five sessions. Note that we only used objects on natural backgrounds because this task was easier compared to previous experiments; therefore, categorizing objects on uniform background would be very easy. For the same reason, we did not used the one-dimension databases in the rapid task.

### 2.3. Behavioral data analysis

We calculated the accuracy of subjects in each experiment as the ratio of correct responses (i.e., Accuracy % = 100 × Number of correct trials / Total number of trials). The accuracies of all subjects were calculated and the average and standard deviation were reported. We also calculated confusion matrices for different conditions of rapid invariant object categorization experiments, which are presented in Supplementary Information. A confusion matrix allowed us to determine which categories were more miscategorized and how categorization errors were distributed across different categories. To calculate the human confusion matrix for each variation condition, we averaged the confusion matrices of all human subjects.

We also analyzed subjects' reaction times in different experiments which are provided in Supplementary Information. In the two-category experiment, first, we removed reaction times longer than 1200 ms (only 7.8% of reaction times were removed across all experiments and subjects). We then compared the reaction times in different experimental conditions. The reported results are the mean and standard deviation of reaction times. In four-category experiments, we removed reaction times longer than 1500 ms because in these tasks it could take longer time to press a key (only 8.7% of reaction times were removed across all experiments and subjects). Although the reaction times in four-category experiments might be a bit unreliable as subjects had to select one key out of four, they provided us with clues about the effect of variations across different dimensions on humans' response time.

### 2.4. Deep convolutional neural networks (DCNNs)

DCNNs are a combination of deep learning (Schmidhuber, 2015) and convolutional neural networks (LeCun and Bengio, 1998). DCNNs use a hierarchy of several consecutive feature detector layers. The complexity of features increases along the hierarchy. Neurons/units in higher convolutional layers are selective to complex objects or object parts. Convolution is the main process in each layer that is generally followed by complementary operations such as pooling and output normalization. Recent deep networks, which have exploited supervised gradient descend based learning algorithms, have achieved remarkable performances in recognizing extensively large and difficult object databases such as Imagenet (LeCun et al., 2015; Schmidhuber, 2015). Here, we evaluated the performance of two most powerful DCNNs (Krizhevsky et al., 2012; Simonyan and Zisserman, 2014) in invariant object recognition. More information about these networks are provided as following:

- **Krizhevsky et al. (****2012****):** This model achieved an impressive performance in categorizing object images from Imagenet database and significantly outperformed other competitors in the ILSVRC-2012 competition (Krizhevsky et al., 2012). Briefly, the model contains five convolutional (feature detector) and three fully connected (classification) layers. The model uses Rectified Linear Units (ReLUs) as the activation function of neurons. This significantly sped up the learning phase. The max-pooling operation is performed in the first, second, and fifth convolutional layers. The model is trained using a stochastic gradient descent algorithm. This network has about 60 millions free parameters. To avoid overfitting during the learning procedure, some data augmentation techniques (enlarging the training set) and the dropout technique (in the first two fully-connected layers) were applied. Here, we used the pre-trained (on the Imagenet database) version of this model (Jia et al., 2014) which is publicly available at http://caffe.berkeleyvision.org.
- **Very Deep (2014):** An important aspect of DCNNs is the number of internal layers, which influences their final performance. Simonyan and Zisserman studied the impact of the network depth by implementing deep convolutional networks with 11, 13, 16, and 19 layers (Simonyan and Zisserman, 2014). For this purpose, they used very small convolution filters in all layers, and steadily increased the depth of the network by adding more convolutional layers. Their results showed that the recognition accuracy increases by adding more layers and the 19-layer model significantly outperformed other DCNNs. Here, we used the 19-layer model which is freely available at http://www.robots.ox.ac.uk/~vgg/research/very_deep/.

### 2.5. Evaluation of DCNNs

We evaluated the categorization accuracy of deep networks on three- and one-dimension tasks with natural backgrounds. To this end, we first randomly selected 600 images from each object category, variation level, and variation condition (three- or one-dimension). Hence, we used 8 different image databases (4 variation levels × 2 variation conditions), each of which consisted of 2500 images (4 categories × 600 images). To compute the accuracy of each DCNN for given variation condition and level, we randomly selected two subsets of 1200 training (300 images per category) and 600 testing images (150 images per category) from the corresponding image database. We then fed the DCNN with the training and testing images and calculated the corresponding feature vectors of the last convolutional layer. Afterwards, we used these feature vectors to train the classifier and compute the categorization accuracy. Here we used a linear SVM classifier (libSVM implementation (Chang and Lin, 2011), www.csie.ntu.edu.tw/~cjlin/libsvm) with optimized regularization parameters. This procedure was repeated for 15 times (with different randomly selected training and testing sets) and the average and standard deviation of the accuracy were computed. This procedure was done for both DCNNs over all variation conditions and levels. Finally, the accuracies of humans and DCNNs were compared in different experiments. For statistical analysis, we used Wilcoxon rank-sum test with α = 0.05. All *p*-values were corrected for multiple comparisons (FDR-corrected, α = 0.05).

To visualize the similarity between the accuracy pattern of DCNNs and human subjects, we performed a Multidimensional Scaling (MDS) analysis across the variation levels of the three-dimension task. For each human subject or DCNN, we put together its accuracies over different variation conditions in a vector. Then we plotted the 2D MDS map based on the cosine similarities (distances) between these vectors. We used the cosine-similarity measure to factor out the impact of mean performance values. Because of the small size of accuracy vectors, correlation-based distance measures were not applicable. Also, contrary to Euclidean distance, the cosine-similarity let us see how the pattern of the accuracy of human subjects and models over different variations are similar or dissimilar, independent of the actual accuracy values.

## 3. Results

We run different experiments in which subjects and DCNNs categorized object images varied across several dimensions (i.e., scale, position, in-plane and in-depth rotations, background). We measured the accuracies and reaction times of human subjects in different rapid and ultra-rapid invariant object categorization tasks, and the effect of variations across different dimensions on human performance was evaluated. Human accuracy was then compared with the accuracy of two well-known deep networks (Krizhevsky et al., 2012; Simonyan and Zisserman, 2014) performing the same tasks as humans. We first report human results in different experiments and then compare them with the results of deep networks.

### 3.1. Human performance is dependent on the type of object variation

In these experiments, subjects were asked to accurately and quickly categorize rapidly presented object images of four categories (car, ship, motorcycle, and animal) appeared in uniform and natural backgrounds (see Section 2.2.1).

**Figure 2:**
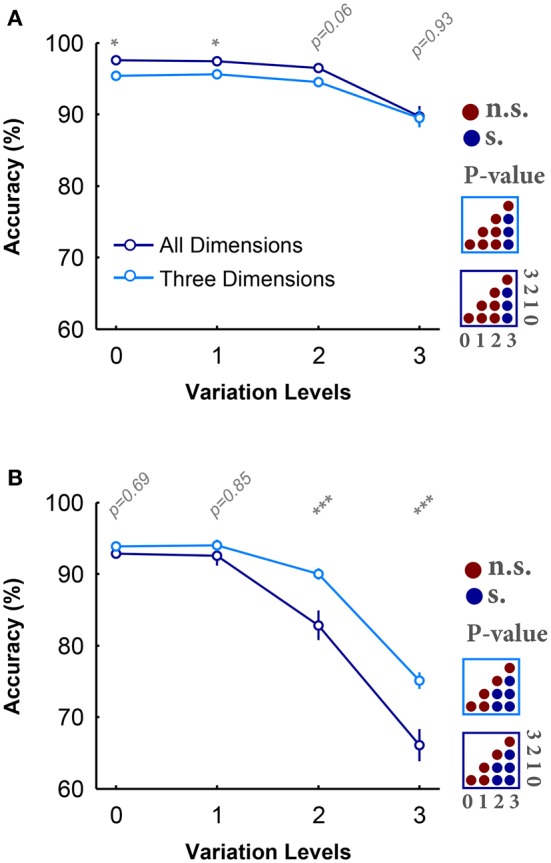
**Accuracy of subjects in rapid invariant object categorization task. (A)** The accuracy of subjects in categorization of four object categories, when objects had uniform backgrounds. The dark, blue curve shows the accuracy when objects varied in all dimensions and the light, blue curve demonstrates the accuracy when objects varied in three dimensions. Error bars are the standard deviation (STD). *P*-values depicted at the top of curves, show whether the accuracy between all- and three-dimension experiment are significantly different (Wilcoxon rank sum test; ^*^P < 0.05, ^**^P < 0.01, ^***^P < 0.001, ^****^P < 0.0001). Color-coded matrices, at the right, show whether changes in accuracy across levels statistically significant (Wilcoxon rank sum test; each matrix corresponds to one curve; see color of the frame). **(B)** Categorization accuracy when objects had natural backgrounds.

Figures 2A,B provide the average accuracy of subjects over different variation levels in all- and three-dimension conditions while objects had uniform and natural backgrounds, respectively. Figure 2A shows that there is a small and negligible difference between the categorization accuracies in all- and three-dimension conditions with objects on uniform background. Also, for both experimental conditions, the categorization errors significantly increased at high variation levels (see the color-coded matrices in the right side of Figure 2A). Despite the small, but significant, accuracy drop, this data shows that humans can robustly categorize object images when they have uniform background even at the highest variation levels (average accuracy above 90%). In addition, the reaction times in all- and three-dimension experiments were not significantly different (Figure S9A).

Conversely, in the case of objects on natural backgrounds (Figure 2B), the categorization accuracies in both experimental conditions substantially decreased as the variation level was increased (see the color-coded matrices in the right side of Figure 2B; Wilcoxon rank sum test), pointing out the difficulty of invariant object recognition in clutter. Moreover, in contrast to the uniform background experiments, there is a large significant difference between the accuracies in all- and three-dimension experiments (see *p*-values depicted at the top of Figure 2B; Wilcoxon rank sum test). Overall, it is evident that excluding one dimension can considerably reduce the difficulty of the task, especially in the natural background case. A similar trend can be seen in the reaction times (see Figure S9B), where the reaction times in both conditions significantly increased as the variation level increased.

**Figure 3:**
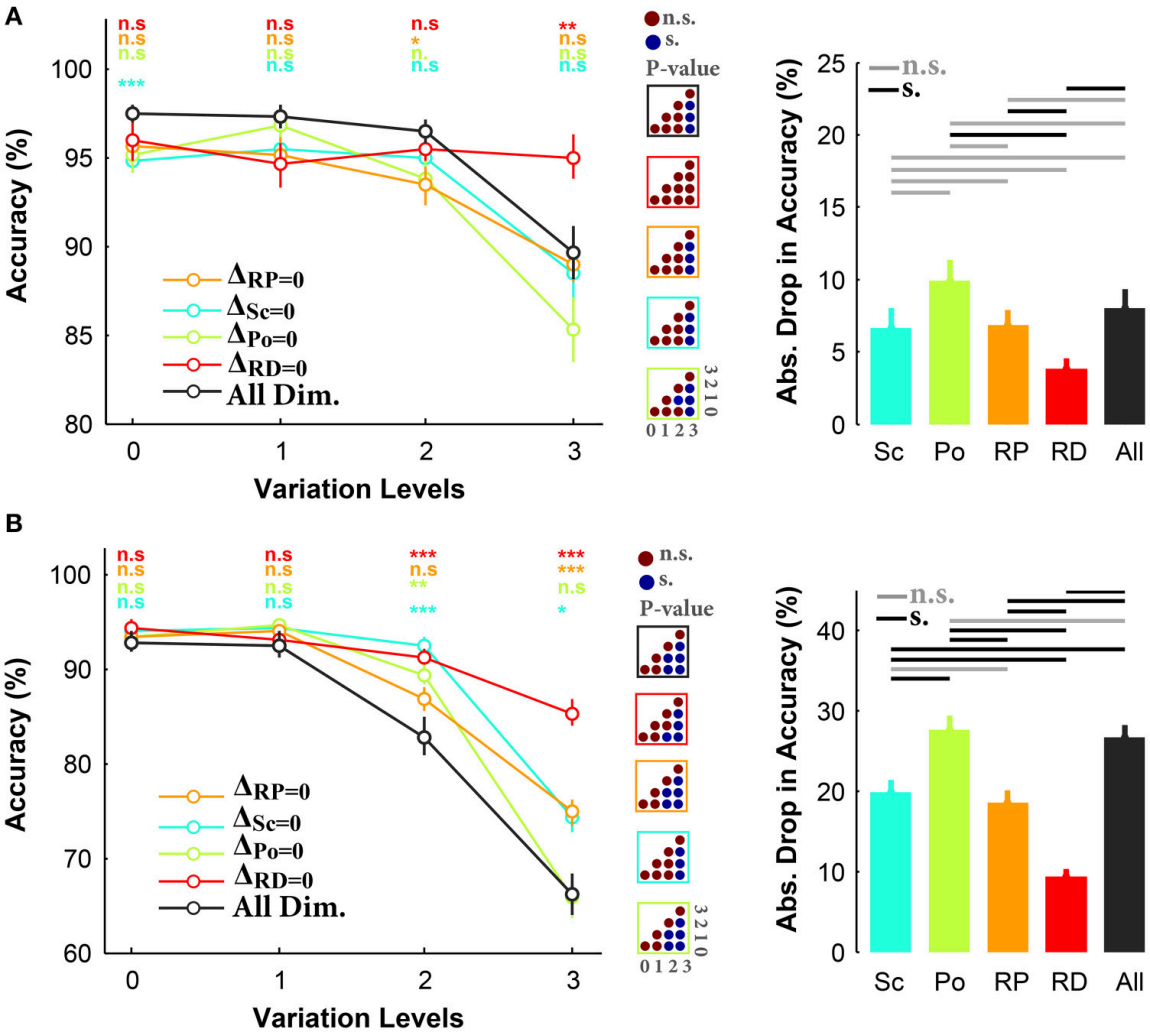
**Accuracy of subjects in rapid invariant object categorization task for all-dimension and different three-dimension conditions. (A)** Accuracies for uniform background experiments. Left, The accuracy of subjects in categorization of four object categories (i.e., car, animal, ship, motorcycle). Each curve corresponds to one condition: Δ_*Sc*_ = 0, Δ_*Po*_ = 0, Δ_*RP*_ = 0, Δ_*RD*_ = 0 (as specified with different colors). Error bars are the standard deviation (STD). *P*-values depicted at the top of curves, show whether the accuracy between all-dimension and other three-dimension conditions are significantly different (Wilcoxon rank sum test; ^*^P < 0.05, ^**^P < 0.01, ^***^P < 0.001, ^****^P < 0.0001, n.s., not significant). Color-coded matrices, at the right, show whether changes in accuracy across levels are statistically significant (e.g., accuracy drop is significant from one level to the other; Wilcoxon rank sum test; each matrix corresponds to one curve; see color of the frame). Right, absolute accuracy drop between level 0 and level 3 (mean+/-STD). The horizontal lines at the top of bar plot shows whether the differences are significant (gray line: insignificant, black line: significant). **(B)** Accuracies for natural backgrounds experiments. Figure conventions are similar to **(A)**.

We then broke the trials into different conditions and calculated the mean accuracy in each condition (i.e., Δ_*Sc*_ = 0, Δ_*Po*_ = 0, Δ_*RP*_ = 0, Δ_*RD*_ = 0). Figure 3A demonstrates the accuracies in all and three-dimension conditions, for the case of objects on uniform background. As seen, there is a small difference in the accuracies of different conditions at low and intermediate variation levels (level 0–2). However, at the highest variation level, the accuracy in Δ_*RD*_ = 0 (red curve) is significantly higher than the other conditions, suggesting that excluding in-depth rotation made the task very easy despite variations across other dimensions. Note that in Δ_*RD*_ = 0 the accuracy curve is virtually flat across levels with average of ~95%. Interestingly, the accuracies were not significantly different between all-dimension experiment and Δ_*Po*_ = 0, Δ_*Sc*_ = 0, and Δ_*RP*_ = 0. This confirms that much of the task difficulty arises from in-depth rotation, although other dimensions have some weaker effects (e.g., scale, and rotation in-plane). This is also reflected in the bar plot in Figure 3A as the absolute accuracy drop in Δ_*RD*_ = 0 is less than 5%, while it is more than 10% in Δ_*Po*_ = 0. It is also clear that humans had the maximum errors in Δ_*Po*_ = 0 condition, suggesting that removing position variation did not considerably affect the task difficulty (i.e., position is the easiest dimension).

The reaction times were compatible with the accuracy results (see Figure S10A), where at the highest variation level, the human reaction times in Δ_*Sc*_ = 0, Δ_*Po*_ = 0, and Δ_*RP*_ = 0 significantly increased, while it did not significantly change in Δ_*RD*_ = 0. In other words, when objects were not rotated in-depth, humans could quickly and accurately categorize them.

In a separate experiment, subjects performed similar task while objects had natural backgrounds. Results show that there were small differences between the accuracies in all- and three-dimension conditions at the first two variation levels (Figure 3B). This suggests that human subjects could easily categorize object images on natural backgrounds while objects had small and intermediate degree of variations. However, accuracies became significantly different as the variation level increased (e.g., levels 2 and 3; see color-coded matrices in Figure 3B). As shown in Figure 3B, there is about 20% accuracy difference between Δ_*RD*_ = 0 and all-dimension condition at the most difficult level, confirming that the rotation in depth is a very difficult dimension. The bar plot in Figure 3B shows that the highest accuracy drop, between levels 0 and 3, belonged to Δ_*Po*_ = 0 and all-dimension conditions while the lowest drop was observed in Δ_*RD*_ = 0. In addition, the accuracies in Δ_*Sc*_ = 0 and Δ_*RP*_ = 0 fall somewhere between Δ_*Po*_ = 0 and Δ_*RD*_ = 0, indicating that scale variations and in-plane rotation imposed more difficulty than variations in position; however, they were easier than rotation in depth. This is also evident in the accuracy drop.

Different objects have different three-dimensional properties; so, the categorization performance might be affected by these properties. In this case, one object category might bias the performance of humans in different variation conditions. To address this question, we broke the trials into different categories and calculated the accuracies (Figure S5) and reaction times (Figures S10B, S11B) for all variation and background conditions. The results indicated that although the categorization accuracy and reaction time may differ between categories, the order of the difficulty of different variation conditions are consistent across all categories. That is, in-depth rotation and position transformation are respectively the most difficult and easy variations to process. We also calculated the confusion matrix of humans for each variation condition and level, to have a closer look at error rate and miscategorization across categories. The confusion matrices for uniform and natural background experiments are presented in Figure S6.

Analyses so far have provided information about the dependence of human accuracy and reaction time on the variations across different dimensions. However, one may ask how these results can be influenced by low-level image statistics such as luminance and contrast. To address this, we computed the correlation between low-level image statistics (contrast and luminance) and the performance of human subjects. The results show that neither luminance (Figure S7) nor contrast (Figure S8) could explain human accuracy and reaction time in our invariant object recognition tasks.

We also performed similar two-category rapid tasks and their results are provided in Supplementary Information (Figures S1–S4). Interestingly, the results of two-category experiments are consistent with the four-category tasks, indicating that our results are robust to the number of categories.

### 3.2. Human performance is independent of experimental setup

Although the effect of variations across different dimensions of an object on subjects' performance was quite robust, we designed two other experiments to investigate how decreasing the presentation time would affect our results. Therefore, we reduced the time of image presentation and the following blank screen from 25 ms to 12.5 ms (ultra-rapid object presentation). We also increased the time of the subsequent noise mask from 100 ms to 200 ms. In the first experiment, we repeated the natural background three-dimension categorization task with the ultra-rapid setting. We did not run uniform background condition because our results showed that this task would be easy and some ceiling effects may mask differences between conditions. For the second experiment, we studied the effect of each individual dimension (e.g., scale only) on the accuracy and reaction time of subjects. In the following, we report the results of these two experiments.

#### 3.2.1. Shorter presentation time does not affect human performance

**Figure 4:**
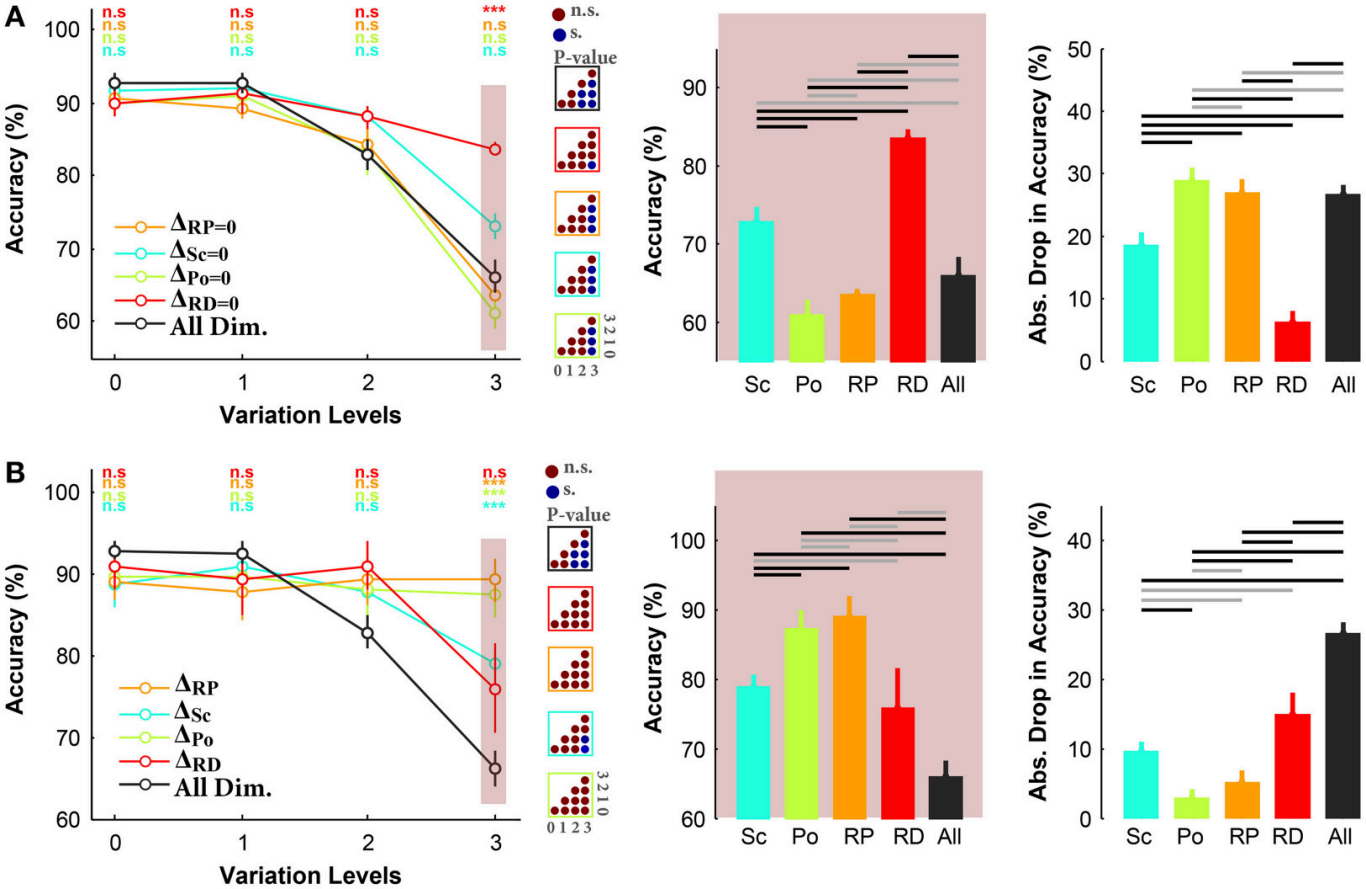
**Accuracy of human subjects in ultra-rapid invariant object categorization task for three- and one-dimension conditions, when objects had natural backgrounds. (A)** Left, the accuracy of human subjects in three-dimension experiments. Each curve corresponds to one condition: Δ_*Sc*_ = 0, Δ_*Po*_ = 0, Δ_*RP*_ = 0, Δ_*RD*_ = 0 (as specified with different colors). Error bars are the standard deviation (STD). *P*-values depicted at the top of curves, show whether the accuracy between all-dimension and other three-dimension conditions are significantly different (Wilcoxon rank sum test; ^*^P < 0.05, ^**^P < 0.01, ^***^P < 0.001, ^****^P < 0.0001, n.s., not significant). Color-coded matrices, on the right show whether changes in accuracy across levels in each condition are statistically significant (e.g., accuracy drop is significant from one level to the other; Wilcoxon rank sum test; each matrix corresponds to one curve; see color of the frame). Note that the results of the average and STD of 5 subjects. Middle, categorization accuracy in level 3 in different three-dimension conditions (each bar corresponds to a condition). The horizontal lines on top of the bar plot shows whether the differences are significant (gray line: insignificant, black line: significant). Right, absolute accuracy drop between level 0 and level 3 (mean+/−STD). Each bar, with specific color, corresponds to one condition. **(B)** Similar to part **(A)**, where the plots present the results in one-dimension experiments.

Figure 4A illustrates the results of the ultra-rapid object categorization task in three-dimension conditions with objects on natural backgrounds. Comparing the results in rapid (see Figure 3B) and ultra-rapid experiments (see Figure 4A, the left plot) indicates that there is no considerable difference between the accuracies in these two experiments. This shows the ability of human visual system to extract sufficient information for invariant object recognition even under ultra rapid presentation.

Similar to the rapid experiment, subjects had the highest categorization accuracy in Δ_*RD*_ = 0 condition, even at the most difficult level, with significant difference to other conditions (see the middle plot in Figure 4A). However, there is a significant difference in accuracies (~10%) between Δ_*Sc*_ = 0 and Δ_*RP*_ = 0. In other words, tolerating scale variation seems to be more difficult than in-plane rotation in ultra-rapid presentation task. It suggests that it is easier to recognize a rotated object in plane than a small object. Comparing the accuracies in level 3 indicates that Δ_*RD*_ = 0 and Δ_*Sc*_ = 0 were the easiest tasks while Δ_*Po*_ = 0 and Δ_*RP*_ = 0 were the most difficult ones. Moreover, although there was no significant difference in reaction times of different conditions (Figure S12A), subjects had shorter reaction times in Δ_*RD*_ = 0 at level 3 while the reaction times were longer in Δ_*Po*_ = 0 at this level.

Overall, the results of ultra-rapid experiment showed that different time setting did not change our initial results about the effect of variations across different dimensions, despite imposing higher task difficulty.

#### 3.2.2. Humans have consistent performances in one-dimension experiment

In all experiments so far, object images varied across more than one dimension. In this experiment, we evaluated the performance of human subjects in ultra-rapid object categorization task while objects varied across a single dimension. Object images were presented on natural backgrounds. Figure 4B illustrates that the accuracies were higher in Δ_*RP*_ and Δ_*Po*_ than in Δ_*RD*_ and Δ_*Sc*_ conditions. Hence, similar to results shown in Figure 4A for three-dimension experiments, variations across position and in-plane rotation were easier to tolerate than in scale and in-depth rotation (again the most difficult). Subjects also had the highest accuracy drop between levels 0 and 3 in Δ_*RD*_ and Δ_*Sc*_ conditions while the accuracy drop in Δ_*RP*_ was significantly lower (bar plots in Figure 4B).

The reaction times in different conditions are shown in Figure S12B. Although the differences were not statistically significant, the absolute increase in reaction time in Δ_*Sc*_ and Δ_*RD*_ was higher than the other conditions, confirming that these variations needed more processing time (note that the results are average of five subjects, and increasing the number of subjects might lead to significant differences).

### 3.3. DCNNs perform similarly to humans in different experiments

We examined the performance of two powerful DCNNs on our three- and one-dimension databases with objects on natural backgrounds. We did not use gray background because it would be too easy for categorization. The first DCNN was the 8-layer network, introduced in Krizhevsky et al. (2012), and the second was a 19-layer network, also known as Very Deep model (Simonyan and Zisserman, 2014). These networks achieved great performance on Imagenet as one of the most challenging current images databases.

**Figure 5:**
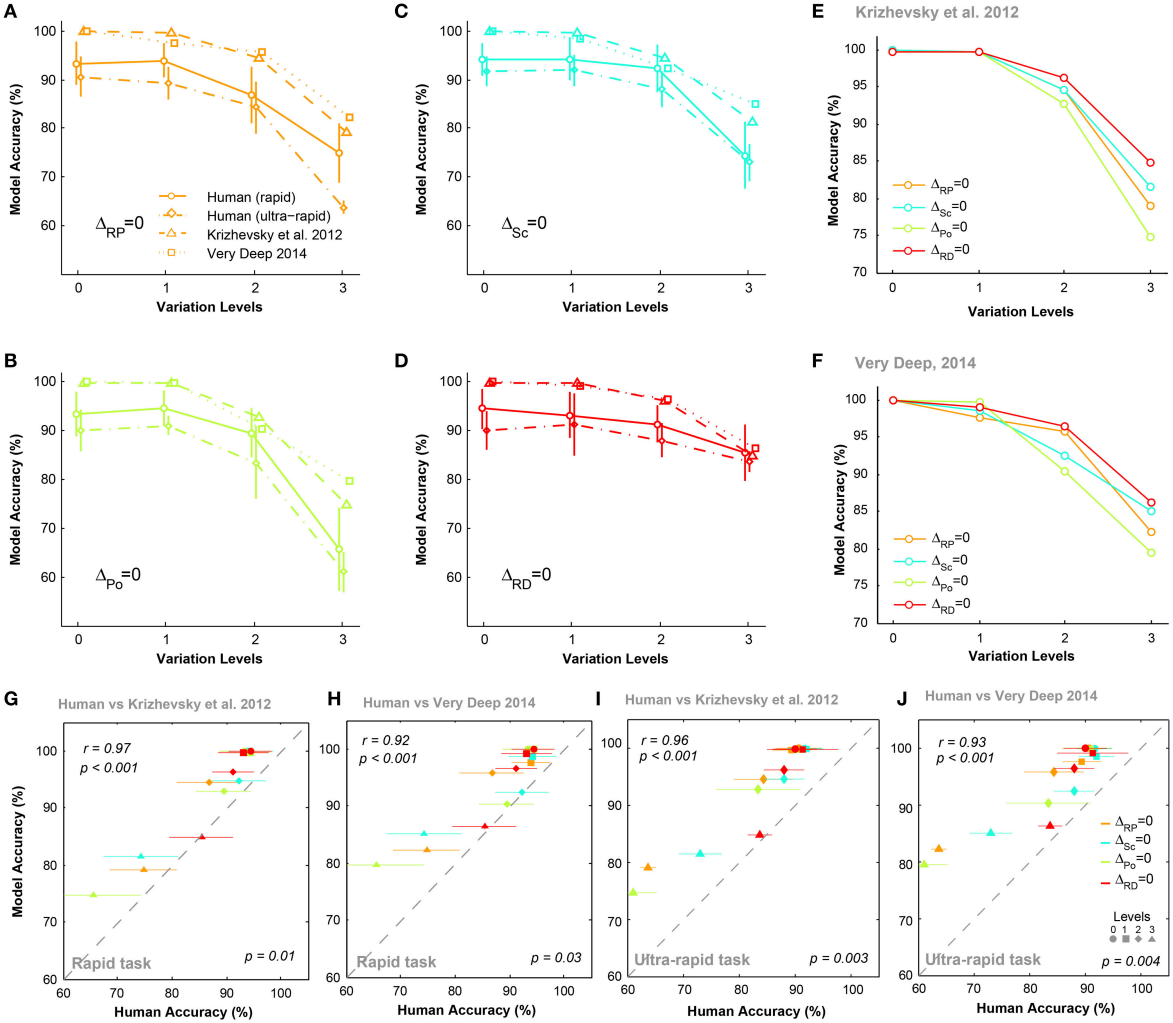
**The accuracy of DCNNs compared to humans in rapid and ultra-rapid three-dimension object categorization tasks. (A–D)** The accuracy of Very Deep (dotted line) and Krizhevsky models (dashed line) compared to humans in categorizing images from three-dimension database while objects had natural background. **(E,F)** The average accuracy of DCNNs in different conditions. **(G,H)** Scatter plots of human accuracy in rapid three-dimension experiment against the accuracy of DCNNs. **(I,J)** Scatter plot of human accuracy in ultra-rapid three-dimension experiment against the accuracy of DCNNs. Colors show different condition and marker shapes refer to variation levels. The correlation is depicted on the upper-left and the *p*-value on lower-right shows whether human and models are significant.

Figures 5A–D compare the accuracies of DCNNs with humans (for both rapid and ultra-rapid experiments) on different conditions for three-dimension databases (i.e., Δ_*Po*_ = 0, Δ_*Sc*_ = 0, Δ_*RP*_ = 0, and Δ_*RD*_ = 0). Interestingly, the overall trend in accuracies of DCNNs were very similar to humans in different variation conditions of both rapid and ultra-rapid experiments. However, DCNNs outperformed humans in different tasks. Despite significantly higher accuracies of both DCNNs compared to humans, DCNNs accuracies were significantly correlated with those of humans in rapid (Figures 5G,H) and ultra-rapid (Figures 5I,J) experiments. In other words, deep networks can resemble human object recognition behavior in the face of different types of variation. Hence, if a variation is more difficult (easy) for humans, it is also more difficult (easy) for DCNNs.

We also compared the accuracy of DCNNs in different experimental conditions (Figures 5E,F). Figure 5E shows that the Krizhevsky network could easily tolerate variations in the first two levels (levels 0 and 1). However, the performance decreased at higher variation levels (levels 2 and 3). At the most difficult level (level 3), the accuracy of DCNNs were highest in Δ_*RD*_ = 0 while this significantly dropped to lowest accuracy in Δ_*Po*_ = 0. Also, accuracies were higher in Δ_*Sc*_ = 0 than Δ_*RP*_ = 0. Similar result was observed for Very Deep model with slightly higher accuracy (Figure 5F).

**Figure 6:**
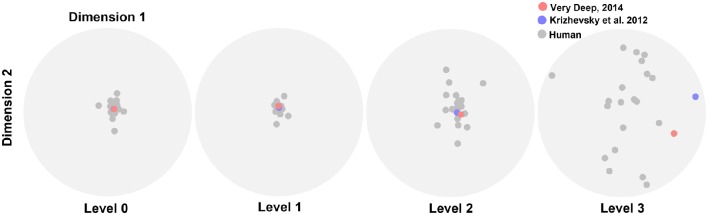
**The similarity between DCNNs and humans**. Scatter plots obtained using multidimensional scaling (MDS). Each plot shows the similarity distances for a variation level. Gray dots illustrate human subjects while red (Very Deep) and blue (Krizhevsky) dots refer to the DCNNs.

We performed the MDS analysis based on cosine-similarity measure (see Materials and methods) to visualize the similarity between the accuracy pattern of DCNNs and all human subjects over different variation dimensions and variation levels. For this analysis, we used the rapid categorization data only (20 subjects), and not the ultra-rapid one (5 subjects only, which is not sufficient for MDS). Figure 6 shows that the similarity between DCNNs and humans is high at the first two variation levels. In other words, there is no difference between humans and DCNNs in low variation levels and DCNNs treat different variations as humans do. However, the distances between DCNNs and human subjects increased at level 2 and became greater at level 3. This points to the fact that as the level of variation increases the task becomes more difficult for humans and DCNNs and the difference between them increases. Although DCNNs get further away from humans, it is not much greater than human inter-subject distances. Hence, it can be said that even in higher variation levels DCNNs perform similarly to humans. Moreover, the Very Deep network is closer to humans than the Krizhevsky model. This might be the result of exploiting more layers in Very Deep network which helps it to act more human-like.

**Figure 7:**
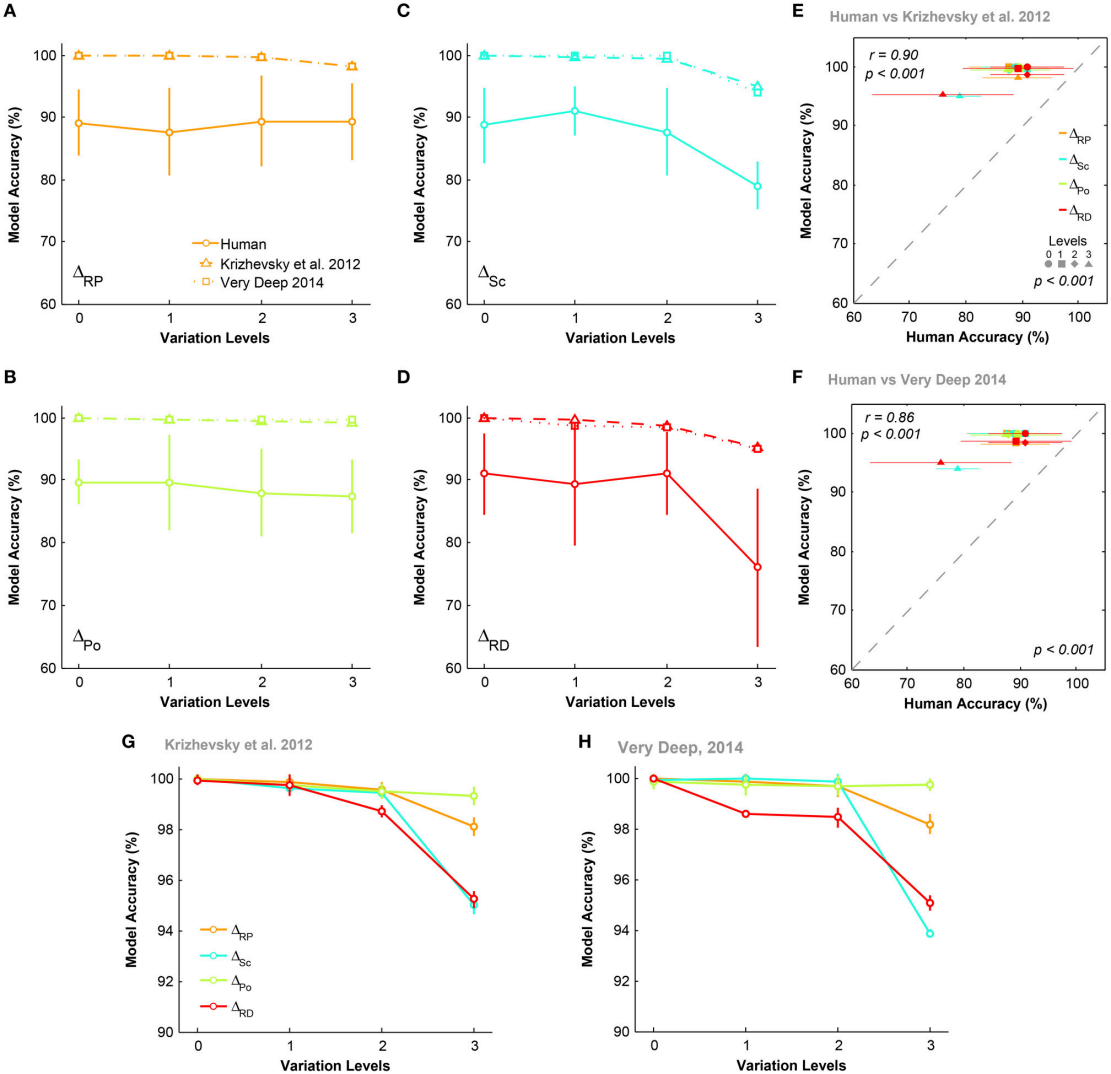
**The accuracy of DCNNs compared to humans in invariant object categorization. (A–D)** The accuracy of Very Deep (dotted line) and Krizhevsky models (dashed line) compared to humans (solid line) in categorizing images from one-dimension database while object had natural background. **(E,F)** Scatter plot of human accuracy against the accuracy of DCNNs. Colors show different condition and marker shapes refer to variation levels. The correlation is depicted on the upper-left and the *p*-value on lower-right shows whether human and models are significant. **(G,H)** The average accuracy of DCNNs in different condition.

To compare DCNNs with humans in the one-dimension experiment, we also evaluated the performance of DCNNs using one-dimension database with natural backgrounds (Figure 7). Figures 7A–D illustrate that DCNNs outperformed humans across all conditions and levels. The accuracies of DCNNs were about 100% at all levels. Despite this difference, we observed a significant correlation between the accuracies of DCNNs and humans (Figures 7E,F), meaning that when a condition was difficult for humans it was also difficult for the models.

To see how the accuracies of DCNNs depend on the dimension of variation, we re-plotted the accuracies of the models in different conditions (Figures 7G,H). It is evident that both DCNNs performed perfectly in Δ_*Po*_, which is possibly inherent by their network design (the weight sharing mechanism in DCNNs Kheradpisheh et al., 2016a), while they achieved relatively lower accuracies in Δ_*Sc*_ and Δ_*RD*_. Interestingly, these results are compatible with humans' accuracy over different variation conditions of one-dimension psychophysics experiment (Figure 4), where the accuracies of Δ_*Po*_ and Δ_*RP*_ were high and almost flat across the levels and the accuracies of Δ_*Sc*_ and Δ_*RD*_ were lower and significantly dropped in the highest variation level.

## 4. Discussion

Although it is well known that the human visual system can invariantly represent and recognize various objects, the underlying mechanisms are still mysterious. Most studies have used object images with very limited variations in different dimensions, presumably to decrease experiment and analysis complexity. Some studies investigated the effect of a few variations (e.g., scale and position) on neural and behavioral responses (Brincat and Connor, 2004; Hung et al., 2005; Zoccolan et al., 2007; Rust and DiCarlo, 2010). It was shown that different variations are differently treated trough the ventral visual pathway, for example, responses to variations in position emerges earlier than variations in scale (Isik et al., 2014). However, there is no data addressing this for other variations. Depending on the type of variation, the visual system may use different sources of information to handle rapid object recognition. Therefore, the responses to each variation, separately or in different combinations, can provide valuable insight about how the visual system performs invariant object recognition. Because DCNNs claim to be bio-inspired, it is also relevant to check if their performance, when facing these transformations, correlates with that of humans.

Here, we performed several behavioral experiments to study the processing of objects that vary across different dimensions through the visual system in terms of reaction time and categorization accuracy. To this end, we generated a series of image databases consisting of different object categories that varied in different combinations of four major variation dimensions: scale, position, in-plane and in-depth rotations. These databases were divided into three major groups: (1) objects that varied in all four dimensions; (2) object that varied in combination of three dimensions (all possible combinations); and (3) objects that varied only in a single dimension. In addition, each database has two background conditions: uniform gray and natural. Hence, our image database has several advantages for studying the invariant object recognition. First, it contains a large number of object images, changing across different types of variation such as geometric dimensions, object instance, and background. Second, we had a precise control over the amount of variations in each dimension which let us generate images with different degrees of complexity/difficulty. Therefore, it enabled us to scrutinize the behavior of humans, while the complexity of object variations gradually increases. Third, by eliminating dependencies between objects and backgrounds, we were able to study invariance, independent of contextual effects.

Different combinations of object variations allowed us to investigate the role of each variation and their combinations in the task complexity and human performance. Interestingly, although different variations were linearly combined, the effects on reaction time and accuracy were not modulated in that way, suggesting that some dimensions substantially increased the task difficulty. The overall impression of our experimental results indicate that humans responded differently to different combination of variations, some variations imposed more difficulty and required more processing time. Also, reaction times and categorization accuracies indicated that natural backgrounds significantly affects invariant object recognition.

Results showed that in-depth rotation is the most difficult dimension either in combination with others or by itself. In case of three-dimension experiments, subjects had high categorization accuracy when object were not rotated in-depth, while their accuracy significantly dropped in other three-dimension conditions. The situation was similar for the reaction times: when the in-depth rotation was fixed across levels, the reaction time was shorter than the other conditions which objects were rotated in-depth. Although we expected that rotation in plane might be more difficult than scale, our results suggest the opposite. Possibly, changing the scale of the object might change the amount of information conveyed through the visual system in which would affect the processing time and accuracy. Besides, the accuracy was very low when the objects were located on the center of the image but varied in other dimensions, while the accuracy was higher when we changed the object position and fixed any other dimensions. This suggests that subjects could better tolerate variations in objects' position.

Moreover, we investigated whether these effects are related to low-level image statistics such as contrast and luminance. The results showed that the correlation between these statistics and reaction time as well as accuracy is very low and insignificant across all levels, type of variations, and objects. This suggests that although different variations affect the contrast and luminance, such low-level statistics have little effect on reaction time and accuracy.

We also performed ultra-rapid object categorization experiments for the three-dimension databases with natural backgrounds, to see if our results depend on presentation condition or not. Moreover, to independently check the role of each individual dimension, we run one-dimension experiments in which objects were varied across only one dimension. These experiments confirmed the results of our previous experiments.

In addition to object transformations, background variation can also affect the categorization accuracy and time. Here, we observed that using natural images as object backgrounds seriously reduced the categorization accuracy and concurrently increased the reaction time. Importantly the backgrounds we used were quite irrelevant. We removed object-background dependency, to purely study the impacts of background on invariant object recognition. However, object-background dependency can be studied in future to investigate how contextual relevance between the target object and surrounding environment would affect the process of invariant object recognition (Bar, 2004; Rémy et al., 2013; Harel et al., 2014).

During the last decades, computational models have attained some scale and position invariance. However, attempts for building a model invariant to 3D variations has been marginally successful. In particular, recently developed deep neural networks has shown merits in tolerating 2D and 3D variations (Cadieu et al., 2014; Ghodrati et al., 2014; Kheradpisheh et al., 2016b). Certainly, comparing the responses of such models with humans (either behavioral or neural data) can give a better insight about their performance and structural characteristics. Hence, we evaluated two powerful DCNNs over the three- and one-dimension databases to see whether they treat different variations as humans do. It was previously shown that these networks can tolerate variations in similar order of the human feed-forward vision (Cadieu et al., 2014; Kheradpisheh et al., 2016b). Surprisingly, our results indicate that, similar to humans, DCNNs also have more difficulties with in-depth rotation and scale variation. It suggests that humans have more difficulty for those variations which are computationally more difficult. Hence, our findings do not argue in favor of three-dimensional object representation theories, but suggests that object recognition can be done mainly based on two-dimensional template matching.

However, there are several studies demonstrating that DCNNs do not solve the object recognition problem in the same way as humans do and can be easily fooled. In Nguyen et al. (2015), authors generated a set of images that were totally unrecognizable for humans, but DCNNs certainty believed that there are familiar objects. Also, in Goodfellow et al. (2014), authors showed that applying a tiny perturbation on input image, which is not noticeable to humans, can drastically decrease the DCNNs performance. Hence, although our results indicate that DCNNs and humans face the same difficulties on different variations, they do not mean that DCNNs also handle these variations similarly. Of course, comparing the internal representation of DCNNs and primates' neural data when they are faced with different object variations can provide a better understanding in this regard.

Moreover, the human visual system extensively exploits feedback and recurrent information to refine and disambiguate the visual representation. Hence, the human visual system would have higher accuracies if it was allowed to use feedback information and continuous visual input. But deep networks lack such mechanisms that could help them to increase their invariance and recognition ability. The future advances in deep networks should put more focus on feedback and continuous vision.

One possible limitation of our work is that we did not assess to what extent previous experience is required for invariant object recognition. Here, presumably humans (trough the development) and DCNNs (through training on Imagenet) had previous experiences with the four classes we used (car, ship, motorcycle, and animal) at different positions, scales, and with different viewing angles, and it is likely that this helped them to develop invariant responses. Certainly, more investigations on how visual experience changes the object representations and neural processing in visual cortex would help to develop more powerful and human-like computational models. For instance, some studies have shown that invariant object representations in visual cortex can be altered through a short learning phase (Cox et al., 2005; Li and DiCarlo, 2008, 2010), and theories like trace learning rule (Földiák, 1991) suggest that temporal association is the key to learning invariance. Applying such learning mechanisms may improve the invariance of DCNNs. Also, another study showed that view-invariance in visual cortex develops later than size-invariance (Nishimura et al., 2015). It would be interesting to perform similar experiments as ours with subjects at different ages, to unravel how invariances to different variations evolve through the development.

Finally, our results showed that variation levels strongly modulate both humans and DCNNs recognition performances, especially for rotation in depth and scale. Therefore, these variations should be controlled in all the image datasets used in vision research. Failure to do so may lead to noisy results, or even misleading ones. For example, in a given dataset, a category may appear easier to recognize than another one only because its variation levels are smaller. We thus think that our methodology and image databases could be considered as benchmarks for investigating the power of any computational model in tolerating different object variations. Such results could then be compared with biological data (electrophysiology, fMRI, MEG, EEG) in terms of performance as well as representational dissimilarity (Kriegeskorte et al., 2008). It would help computational modelers to systematically evaluate their models in fully controlled invariant object recognition tasks, improve the variation tolerance in their models, and make them more human-like.

## Author contributions

SK, MGh, MG, and TM sketched the experiments. SK and MGh performed the experiments and wrote the first draft of the manuscript. SK, MGh, MG, and TM contributed to the analysis of data and the preparation of manuscript.

### Conflict of interest statement

The authors declare that the research was conducted in the absence of any commercial or financial relationships that could be construed as a potential conflict of interest.
