# Supplementary material for: Humans and Deep Networks Largely Agree on Which Kinds of Variation Make Object Recognition Harder
Source: Front Comput Neurosci. 2016 Aug 31;10:92. doi: 10.3389/fncom.2016.00092 (PMC5015476; doi:10.3389/fncom.2016.00092)
Supplement: Supplementary file 1 [file Presentation1.PDF]

Supplementary Material:
Humans and deep networks largely agree on which kinds of variation make object recognition harder

Saeed Reza Kheradpisheh, Masoud Ghodrati, Mohammad Ganjtabesh and Timothée Masquelier

*Correspondence:
Mohammad Ganjtabesh
mgtabesh@ut.ac.ir

1 TWO-CATEGORY RAPID PRESENTATION EXPERIMENTS

1.1 Psychophysics experiment

In these experiments, subjects categorized rapidly presented images from two categories: car and animal. Each trial started with a fixation cross presented at the center of the screen for 500 ms. An image was then randomly selected from the image database and was presented for 25 ms (2 frames of a 80 Hz monitor) followed by a gray blank screen for 25 ms (ISI). Immediately after the blank screen, a $1/f$ noise mask image was presented for 100 ms. Subjects were asked to rapidly and accurately press one of the two keys, labeled on the computer keyboard, to indicate which object category was presented. The next trial was started immediately after the key press. There were two conditions which are explained as following:

- **All-dimension database:** Object images were selected from the all-dimension database (see Image generation in the main manuscript). Subjects participated into two sessions: 1) Objects on a gray uniform background; 2) Objects on randomly selected natural backgrounds. In each session, subjects were presented with 320 object images (2 categories $\times$ 4 levels $\times$ 40 images from each category), divided into two blocks of 160 images. We collected the data of 17 sessions for each condition (i.e., uniform and natural backgrounds).
- **Three-dimension database:** In this experiment, we used the three-dimension databases.This allowed us to study the effect of excluding the variations across one dimension on human performance in invariant object categorization: if the fixed dimension is more difficult than the others, subjects will be able to categorize the objects more accurately and within shorter time than if the fixed dimension is easier. In each session, subjects were presented with 960 images: 2 categories $\times$ 4 levels $\times$ 4 conditions ($\Delta_{Sc} = 0$, $\Delta_{Po} = 0$, $\Delta_{RP} = 0$, and $\Delta_{RD} = 0$) $\times$ 30 images per category. Note that we inter-mixed images of all conditions; so, subjects were unaware of the type of variations. Images were presented in four consecutive blocks of 240 images. We recorded 17 sessions for each background condition (i.e., objects on uniform and natural backgrounds).

1.2 Behavioral results

Subjects achieved remarkably high accuracy in categorization of rapidly presented object images from two categories while they varied across different dimensions (car versus animal; see the experimental

settings in previous Section). When objects had uniform background, the average accuracy of subjects across different variation levels was about 95% (Figure S1.A). There was no significant difference between the accuracies when objects varied across all and three dimensions (Wilcoxon rank-sum test). Also, there was no significant accuracy drop across the variation levels in both experimental conditions (see the color-coded p-value matrices at the right side of Figure S1.A, Wilcoxon rank sum test).

However, in the experiment with natural backgrounds, there was a significant accuracy drop as the variation level increased in both all- and three-dimension conditions (see the color-coded p-value matrices in the right side of Figure S1.B). This shows that the presence of distractors in the background dramatically affects the accuracy of subjects in invariant object recognition, specifically at higher variation levels.

Figure S1 showed the overall accuracy in two experimental conditions, but it did not show how accuracy depends on variations across different dimensions. For this purpose, we computed the average accuracies for different conditions in the three-dimension experiment (i.e., $\Delta_{Sc} = 0$, $\Delta_{Po} = 0$, $\Delta_{RP} = 0$, $\Delta_{RD} = 0$) and compared them with the all-dimension case. Figure S2.A shows that when objects had uniform background, there was no significant difference in the accuracies of different three-dimension conditions, suggesting that subjects could robustly categorize objects in this case, even at high variation levels. The accuracy drop between level 0 and level 3 was also very small (see the bar plot in Figure S2.A).

The situation was completely different when objects had natural backgrounds. Figure S2.B illustrates that although there is no significant difference in accuracies between three-dimension conditions at low and intermediate variation levels, there is a significant difference (almost 15%) between the accuracies in $\Delta_{RD} = 0$ and $\Delta_{Sc} = 0$, and $\Delta_{RP} = 0$ and $\Delta_{Po} = 0$ at the highest variation level (level 3). This is also evident in the absolute accuracy drops, which indicates that the accuracy drop in $\Delta_{RD} = 0$ and $\Delta_{Sc} = 0$ was significantly smaller than $\Delta_{RP} = 0$ and $\Delta_{Po} = 0$ (Figure S2.B, bar plot). These suggest that the presence of in-depth rotation and scale variation made the object recognition very difficult. The accuracy in all-dimension experiment was similar to $\Delta_{RP} = 0$ and $\Delta_{Po} = 0$ conditions (See p values at the top of Figure S2).

We also recorded the reaction times of subjects performing two-category experiments. Here, we first report the overall reaction time of human subjects regardless of the type of variations. Figures S3.A and S3.B present the reaction times of different variation levels when objects varied in all and three dimensions for uniform and natural backgrounds, respectively.

The average reaction time in the all-dimension condition is longer than in the three-dimension condition across all levels, although the differences are not significant (Figure S3.A, wilcoxon rank sum). In addition, for both all- and three-dimension condition, there is no significant change in reaction times across the variation levels (see the color-coded p-value matrices in the right side of Figure S3.A; they show all possible pair-wise comparisons across levels; wilcoxon rank sum test). As shown in Figure S1.A, results for the accuracies are similar. Hence, it can be said that humans can accurately (95%) and quickly ($450 - 520$ ms) categorize varied object images in our two-category invariant object categorization tasks with uniform background.

The general reaction times for natural background condition are provided in Figure S3.B. As can be seen, there are significant increments in reaction times of both all- and three-dimension, specifically in higher variation levels. The trend for the accuracies is similar(see Figure S1.B): the accuracies significantly drop in higher variation levels. These together show that distractors in clutter backgrounds significantly affect the performance of humans in recognition of highly varied objects. Moreover, although there was no significant difference in the accuracies between all- and three-dimension experiments, the reaction

times were significantly different at variation levels 2 and 3, indicating that objects with variations across all-dimensions needed more processing time than in the three-dimension case.

Figures S4.A and S4.B demonstrate the reaction times of each variation combination in the three-dimension case as well as in the all-dimension case across different levels for both uniform and natural backgrounds, respectively. Comparing the reaction times in different three-dimension conditions with uniform background shows insignificant difference among them (Figure S4.A). It means that the elimination of any dimension dose not affect much the reaction time. In other words, it is easy for humans to categorize rapidly presented car and animal images with uniform gray background even in high variation levels independently of the type of variations. However, we can see, from the left bar plot, that removing rotation in-depth (red bar) made the task easier with smaller absolute drop in reaction time from level 0 to level 3 (this is significant comparing to other conditions, see color-coded horizontal lines at the top of the bar plots). It is also evident that the all-dimension case has a higher reaction time and it is the most difficult task.

However, as shown in Figure S4.B, the situation is different when objects had natural backgrounds. The reaction times of $\Delta_{RP} = 0$ and $\Delta_{Po} = 0$ are longer than $\Delta_{RD} = 0$ and $\Delta_{Sc} = 0$. As seen in Figure S2.B, the accuracies of $\Delta_{RP} = 0$ and $\Delta_{Po} = 0$ were also significantly lower than that of the other two three-dimension conditions. This confirms that in-depth rotation and scale variations are more difficult than the other two and need more processing time (Figure S4.B). It is also noteworthy that the reaction times in the all-dimension condition are significantly longer than in all the three-dimension conditions (See p values at the top of Figure S4.B).

2 FOUR-CATEGORY RAPID PRESENTATION EXPERIMENTS

2.1 Category-wise accuracy

The global accuracies of humans for the four-category rapid experiments are presented in the main manuscript. Here, for each category, we present the human accuracies in different variation and background conditions. Figure S5.A illustrates the category-wise accuracies in case of uniform background. As seen, the $\Delta_{RD} = 0$ condition has the highest accuracy in almost all categories, even at the highest variation level, while $\Delta_{Po} = 0$ has the lowest accuracy. These two conditions also have the lowest and highest accuracy drops, respectively. Generally, subjects made the maximum error while categorizing motorcycle and ship categories, specifically at the highest variation level, while they achieved the greatest accuracy in categorization of car images. This is also reflected in the accuracy drop bar plots with the highest drop in categorization of motorcycle images and the lowest drop for car images. This means that subjects could better tolerate variations in car images and they have more difficulty to deal with variations in motorcycle and ship instances.

Figure S5.B represents the accuracies of each category in a separate plot, for the natural background experiment. Although the trend in accuracy is different across categories, it is evident that $\Delta_{Sc} = 0$ and $\Delta_{RD} = 0$ were the easiest in three-dimension on conditions. Moreover, subjects had higher errors (and longer reaction times; see Figure S11.B) in categorization of ships and motorcycles compared to the other categories. This can be seen in the bar plots of Figure S5.B, where the highest accuracy drop was observed when categorizing images from the motorcycle category. In contrast, we observed the lowest accuracy drop when categorizing car images. This indicates that, contrary to motorcycle and ship categories, subjects could better tolerate variations in cars. The most difficult categorization task was when position variation

was set to 0 (green curves and bars). In this case, objects varied in the dimensions (i.e., scale, in-depth and in-plane rotations) that imposed more difficulty to the task.

2.2 Confusion matrix analysis

To have a closer look at error rate and miscategorization across categories, we calculated the confusion matrices of all- and three-dimension experiments. Figure S6.A shows that in the uniform background condition, the categorization error increased at level 3, with the highest error rate when categorizing ship and motorcycle images (e.g., most of wrongly assigned ship labels corresponded motorcycles images). Comparing different three-dimension conditions shows that the miscategorization rate in $\Delta_{Po} = 0$ was higher than in the other conditions while the lowest miscategorization rate was observed in $\Delta_{RD} = 0$. The miscategorization rate in natural backgrounds experiment was higher than in the uniform background condition (Figure S6.B), even at low and intermediate variation levels. The error rate was lower in $\Delta_{RD} = 0$ than in the other conditions. On the other hand, the highest error rate was observed in $\Delta_{Po} = 0$.

2.3 Contrast and luminance analysis

As a control, we computed the correlation between low-level image statistics (contrast and luminance) and the performance of human subjects. Here, we investigate whether the changing pattern of human accuracy is due to the nature of the task or to the changes in image statistics. The image contrast is computed by the Root Mean Square Contrast method which is defined as the standard deviation of the intensity values of all pixels in the image divided by the mean intensity, and the image mean luminance is obtained by averaging the pixel intensities.

Figures S7.A and S7.B, respectively, illustrate the correlation values of image contrast and mean luminance with human accuracy and reaction time over different variation levels and variation conditions for the three-dimension uniform background task. As can be seen, the correlation of image contrast with human accuracy and reaction time are negligible and insignificant for almost all variation conditions. The situation is similar for image mean luminance. These together indicate that, for the uniform background task, image statistics such as contrast and luminance do not significantly contribute to humans' invariant object recognition process.

We did the same analysis for the three-dimension natural background conditions. The correlation values of image contrast and mean luminance with human accuracy and reaction time over different variation levels and variation conditions are presented in Figure S8.A and Figure S8.B, respectively. As shown in these figures, the correlation values for both contrast and mean luminance are small and statistically insignificant for all variation levels and variation conditions. This means that changes in luminance or contrast do not affect human accuracy and reaction time in invariant object recognition.

2.4 Reaction time

Figures S9.A and S9.B demonstrate the average reaction time of human subjects over different variation levels of all- and three-dimension experiments, for uniform and natural backgrounds, respectively. Note that in these figures the reaction time of three-dimension conditions are averaged over different variation types, meaning that we did not break into the type of variation.

As seen in Figure S9.A, in case of uniform background the reaction times of the all- and three-dimension experiments are very close to each other and there is no significant difference between these conditions. However, humans needs more processing time ($\sim$ 50ms) for the same tasks but with natural backgrounds (see Figure S9.B). Contrary to the uniform background case, there is a big difference (although not

statistically significant) between the average reaction times of the all- and three-dimension experiments with natural backgrounds.

Figure S10.A provides the reaction times of each three-dimension condition as well as of the all-dimension experiment, for the case of uniform background. In low and intermediate variation levels, the reaction times of the different three-dimension conditions as well as of the all-dimension one are closed to each other. However, in the most difficult level, the reaction times of $\Delta_{Sc} = 0$, $\Delta_{Po} = 0$, and $\Delta_{RP} = 0$ significantly grows up, while it does not change in $\Delta_{RD} = 0$ (see color-coded matrices). Indeed, when an object is not rotated in-depth humans can more quickly categorize it than when another dimension is fixed and the object is allowed to rotate in-depth. This again indicates that humans need more processing time to categorize depth-rotated objects. Evidently, $\Delta_{Po} = 0$ has higher reaction times than the other conditions, specifically in the highest level. In other words, if we do not change the position of the object but vary it in other dimensions, subjects need more time to categorize it. This means that position variation is easier to overcome for humans. Also, these results are confirmed by comparing the absolute increase in reaction times of different conditions presented in the bar plot of Figure S10.A.

For each object category, the reaction times of the all- and three-dimension uniform background experiments are presented in a separate plot in Figure S10.B. In line with categorization accuracies (see Figure S5.A), $\Delta_{RD} = 0$ has the shortest and $\Delta_{Po} = 0$ has the longest reaction times in almost all categories. By the way, Motorcycles have the highest reaction times specifically in level 3, and also have the greatest increase in reaction time. Figure S11.A illustrates the reaction time and absolute increase in reaction time of the all- and three-dimension experiments with natural backgrounds. The category-wise reaction times and corresponding reaction time increments from the lowest to the highest variation level are shown in Figure S11.B.

3 FOUR-CATEGORY ULTRA-RAPID PRESENTATION EXPERIMENTS (REACTION TIME)

The accuracies of humans for these experiments are available in the main manuscript, and here we present the reaction times. The left plot in Figure S12.A illustrates the reaction times of the ultra-rapid invariant object categorization task for the three-dimension conditions, when objects had natural backgrounds. The absolute reaction time increase, from the first to the last variation level, as well as the reaction time in level 3 are also presented in the middle and the right plot of Figure S12.B, respectively. Although there is no statistically significant difference in the reaction times of different conditions, $\Delta_{RD} = 0$ has the lowest average reaction time in level 3 (note that these results are the average of five subjects only, so, small sample size might be the reason for insignificant differences). Also, $\Delta_{RD} = 0$ has the smallest increase in reaction time from the first the highest level. Once again, $\Delta_{Po} = 0$ has the highest average reaction time (although insignificant) in level 3, and the largest increase in reaction time from level 0 to level 3.

The reaction times in different conditions of the one-dimension natural background experiments are also shown in the left plot of Figure S12.B. Although the differences are not statistically significant, the absolute increase in reaction time in Δ_{Sc} and Δ_{RD} is higher than in the other conditions, confirming that these variations need more processing time (note that the results are the average of five subjects only and increasing the sample size might lead to observe significant differences). In addition, $\Delta_{Po} = 0$ and Δ_{RP} has the lowest reaction time increment (see the right plot of Figure S12.B), meaning that these variations need less processing time in the human visual system.

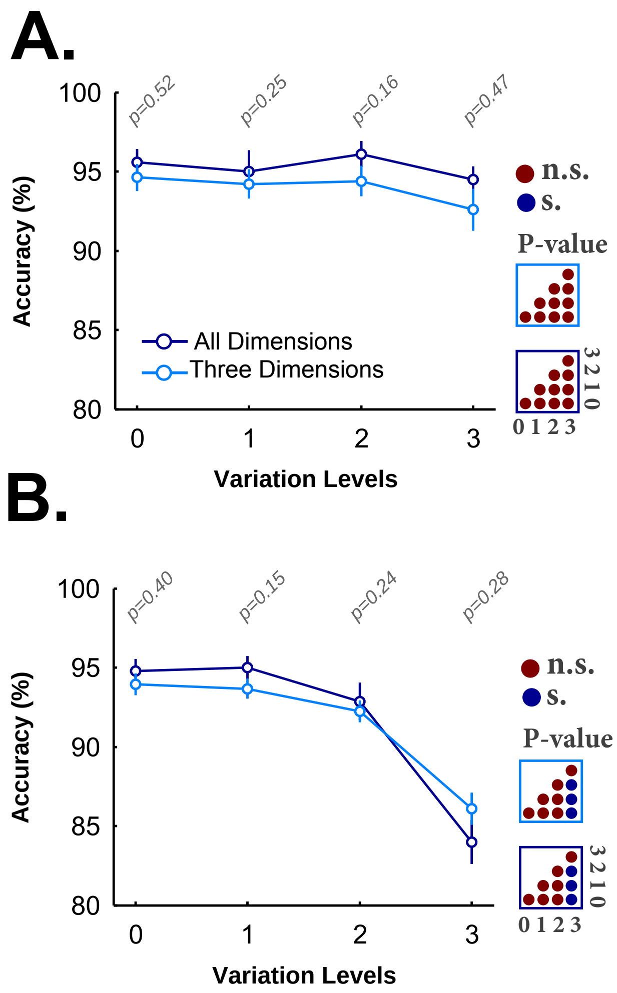

Figure S1. Human accuracy in two-category rapid invariant object categorization task. A. The average accuracy of human subjects in categorization of car versus animal images, when objects had uniform background. The dark, blue curve shows the accuracy when objects varied across all dimensions and the light, blue curve demonstrates the accuracy when objects varied across three dimensions. Error bars are the standard deviation (STD). P values, printed at the top of curves, show whether the accuracy between all- and three-dimension experiments significantly differ (Wilcoxon rank sum test). Color-coded matrices, at the right, show all possible pair-wise comparisons across levels, indicating whether changes in accuracy were statistically significant (Wilcoxon rank sum test; each matrix corresponds to one curve; see color of the frame). B. Categorization accuracies when objects had natural backgrounds.

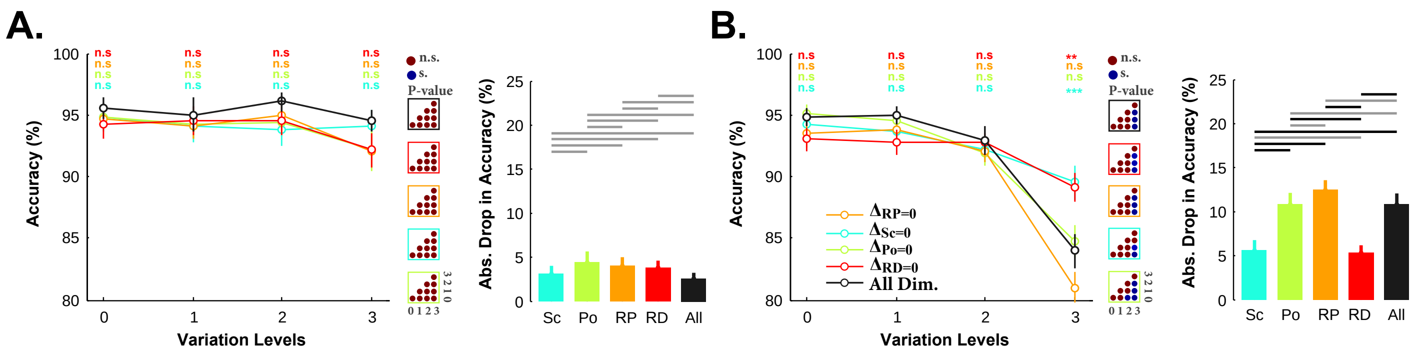

Figure S2. Human accuracy in two-category rapid invariant object categorization task in different three-dimension conditions. A. Left, human average accuracy in categorization of car versus animal images, when objects had uniform backgrounds. Each curve corresponds to one condition: $\Delta_{Sc} = 0$, $\Delta_{Po} = 0$, $\Delta_{RP} = 0$, $\Delta_{RD} = 0$ (as specified with different colors). Error bars are the standard deviation (STD). P values, depicted on the top of curves, show whether the accuracy between all-dimension and three-dimension conditions significantly differ (Wilcoxon rank sum test). Color-coded matrices show whether changes in accuracy across levels are statistically significant (Wilcoxon rank sum test; each matrix corresponds to one curve; see color of the frame). Right, absolute accuracy drop between level 0 and level 3 (mean+/-STD). Each bar corresponds to one condition. The horizontal lines on the top of bar plot show whether the differences are significant (gray line: insignificant, black line: significant). B. Categorization accuracy when objects had natural backgrounds.

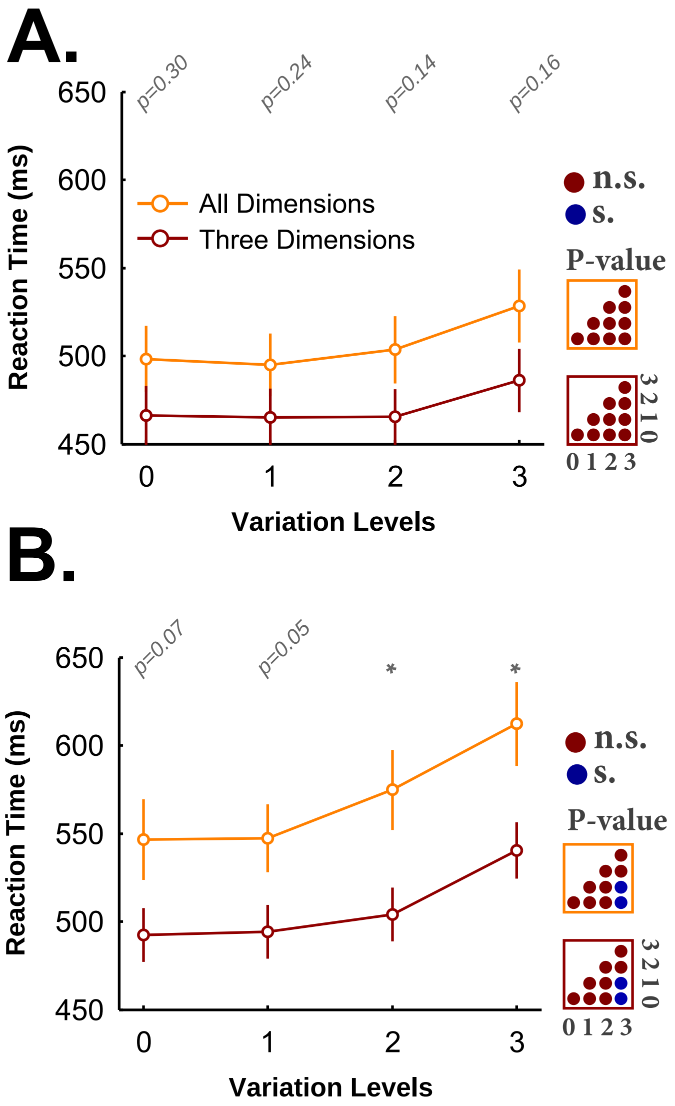

Figure S3. Average reaction time of humans in two-category rapid invariant object categorization task. A. Average and standard error of the mean (SEM) of subjects' reaction time in all- and three-dimension conditions, when objects had uniform background. The orange curve (resp. brown) shows the reaction time when objects varied in all (resp. three) dimensions. Note that reaction times in three-dimension case are the overall reaction times across different conditions. P values, depicted at the top of curves, show whether the reaction time difference between all- and three-dimension are significant (Wilcoxon rank sum test). Color-coded matrices, at the right, show all possible pair-wise comparisons across levels, indicating that whether or not reaction time changes are statistically significant (Wilcoxon rank sum test; each matrix corresponds to one curve; see color of the frame) B. Reaction times when objects had natural background.

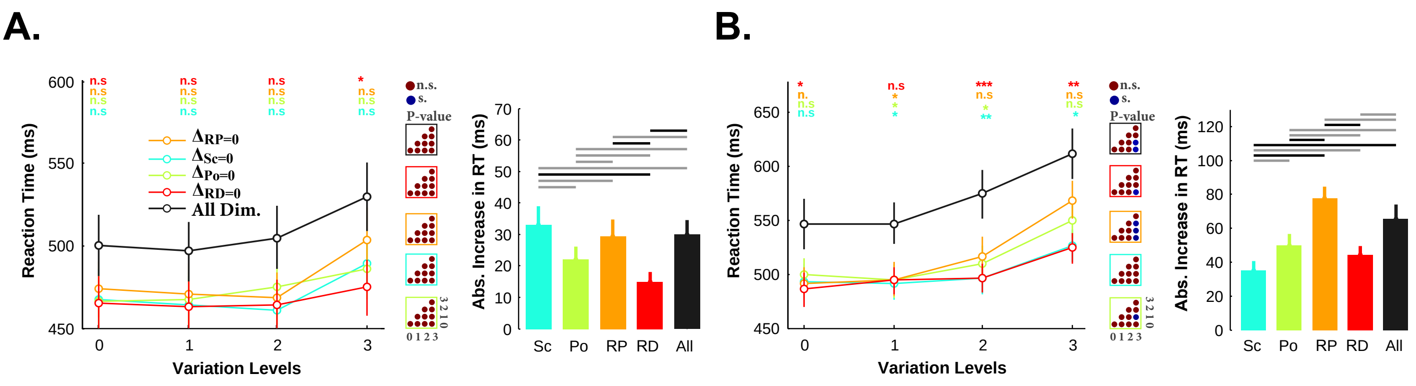

Figure S4. Average reaction time of humans in two-category rapid invariant object categorization task for different three-dimension conditions. A. Left, average and standard error of the mean (SEM) of subjects' reaction time in all-dimension and different three-dimension conditions, when objects had uniform background. Each curve corresponds to one condition: $\Delta_{Sc} = 0$, $\Delta_{Po} = 0$, $\Delta_{RP} = 0$, $\Delta_{RD} = 0$ (as specified with different colors). P values, depicted on the top show whether the reaction time difference between all-dimension and other three-dimension conditions are significant (Wilcoxon rank sum test). Color-coded matrices show all possible pair-wise comparisons across levels, indicating whether or not the reaction time changes in each condition are statistically significant (Wilcoxon rank sum test; each matrix corresponds to one curve; see color of the frame). Right, absolute reaction time increase between level 0 and level 3 (mean+/-STD). The horizontal lines on the top show whether the differences are significant (gray line: insignificant, black line: significant). B. Average and SEM of subjects' reaction time in all- and different three-dimension conditions, when objects had natural backgrounds.

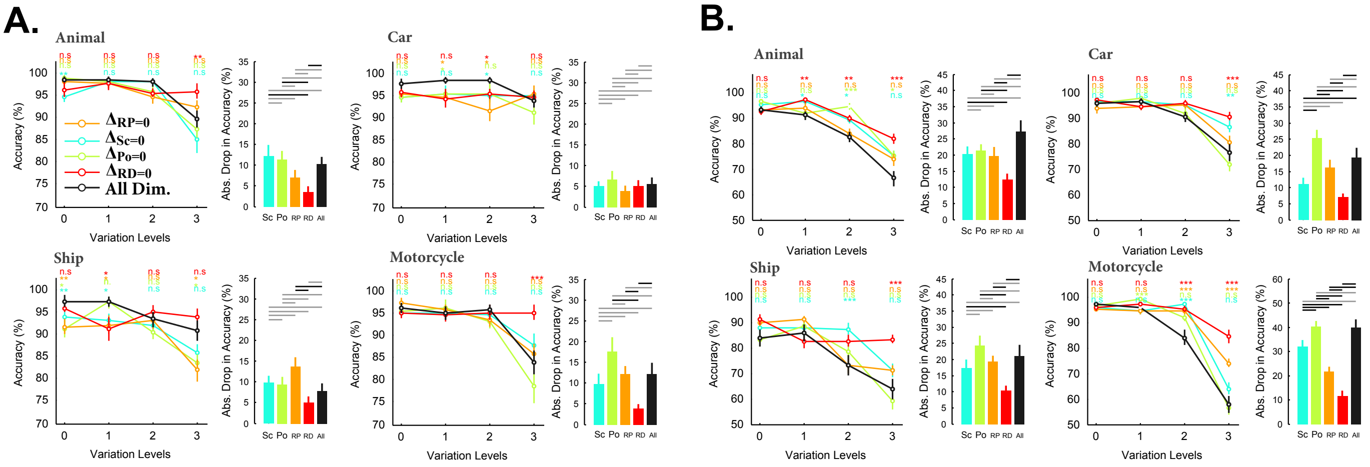

Figure S5. Categorization accuracy and absolute drop in accuracy for different conditions and object categories. A. Uniform background. The left plot for each object category illustrates the accuracies of different three-dimension conditions. Error bars are the standard deviation (STD). P values, depicted on the top show whether the accuracy between all-dimension and other three-dimension conditions are significantly different (Wilcoxon rank sum test). For each object category, the bar plot on the right demonstrates the absolute accuracy drop between level 0 and level 3 (mean+/-STD). The horizontal lines on the top of these bar plots show whether the differences between variation conditions are significant (gray line: insignificant, black line: significant). B. Natural background, the conventions are identical to A.

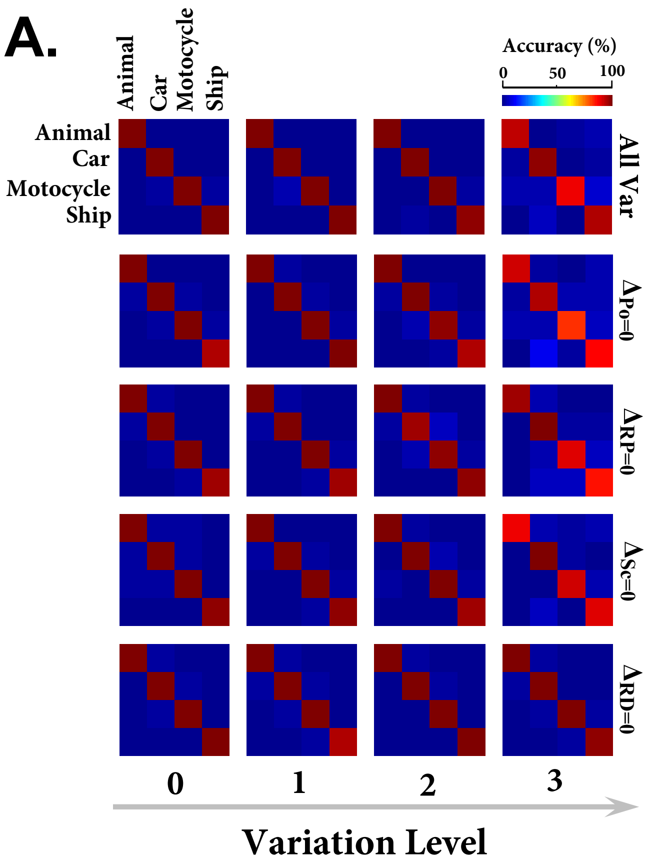

Figure S6. Confusion matrices for rapid object categorization tasks for all- and three-dimension conditions. A. Confusion matrices when objects had uniform backgrounds. Each column of confusion matrices corresponds to a variation level and each row refers to an experimental condition (written at the right end). The name of categories is written at the first, top-left confusion matrix. The color bar at the top-right indicates the range of accuracies. B. Confusion matrices when object has natural backgrounds.

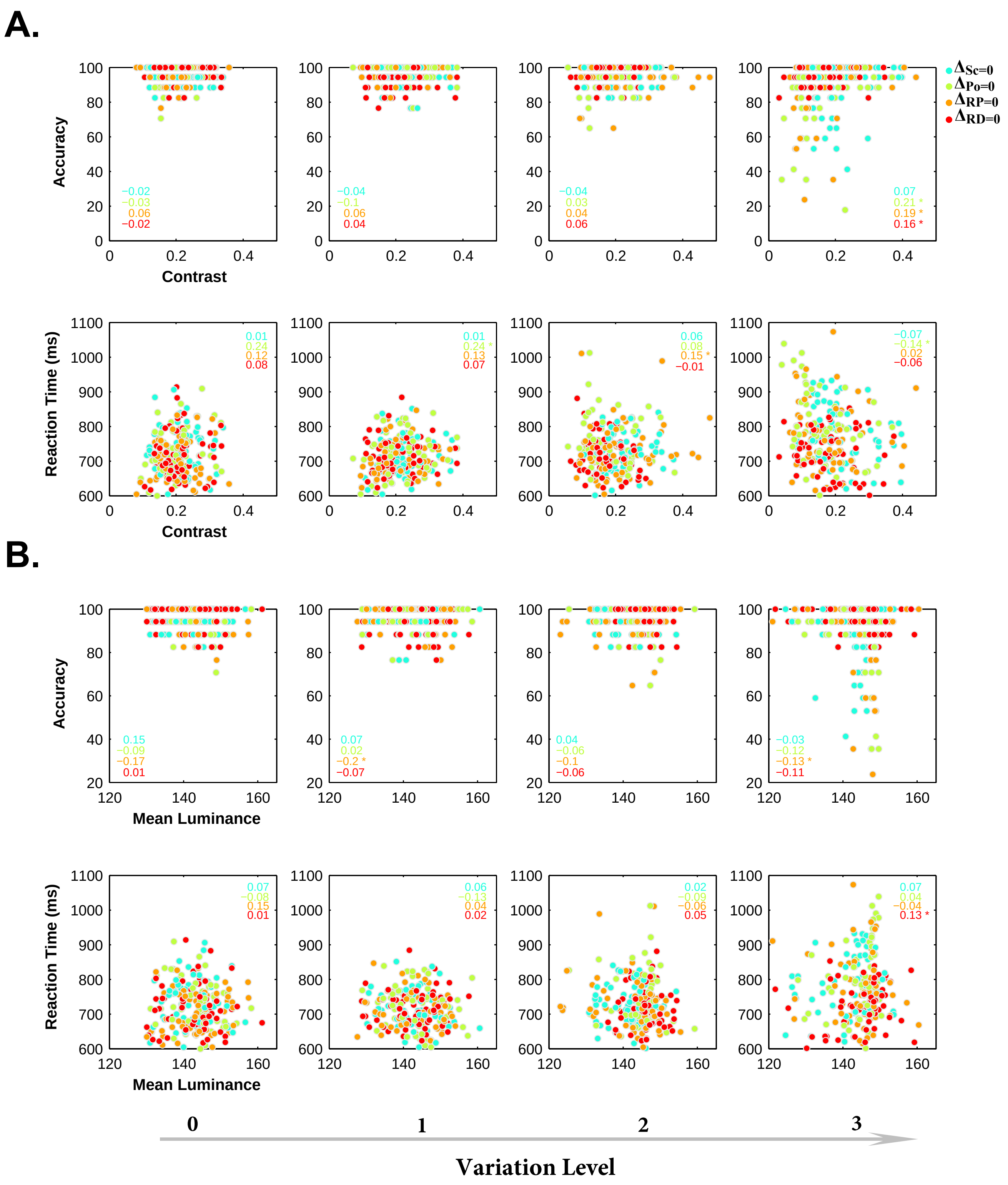

Figure S7. The correlation between low-level statistics (contrast and luminance) and the performance of human subjects, when objects had uniform backgrounds. A. Correlation between the contrast of images (root mean square contrast) and human accuracy (top row) and reaction time (bottom row) for all levels and three variation conditions. Each point refers to an image and colors indicate an experimental condition. Correlation values are depicted in each scatter plot with corresponding colors (Pearson correlation). Significant correlations are specified using asterisks next to numbers. Scatter plots are plotted for all levels from level 0 (left) to level 1 (right). B. Correlation between the luminance of images (mean luminance of all pixels) and human accuracy (top row) and reaction time (bottom row) for all levels and three variation conditions.

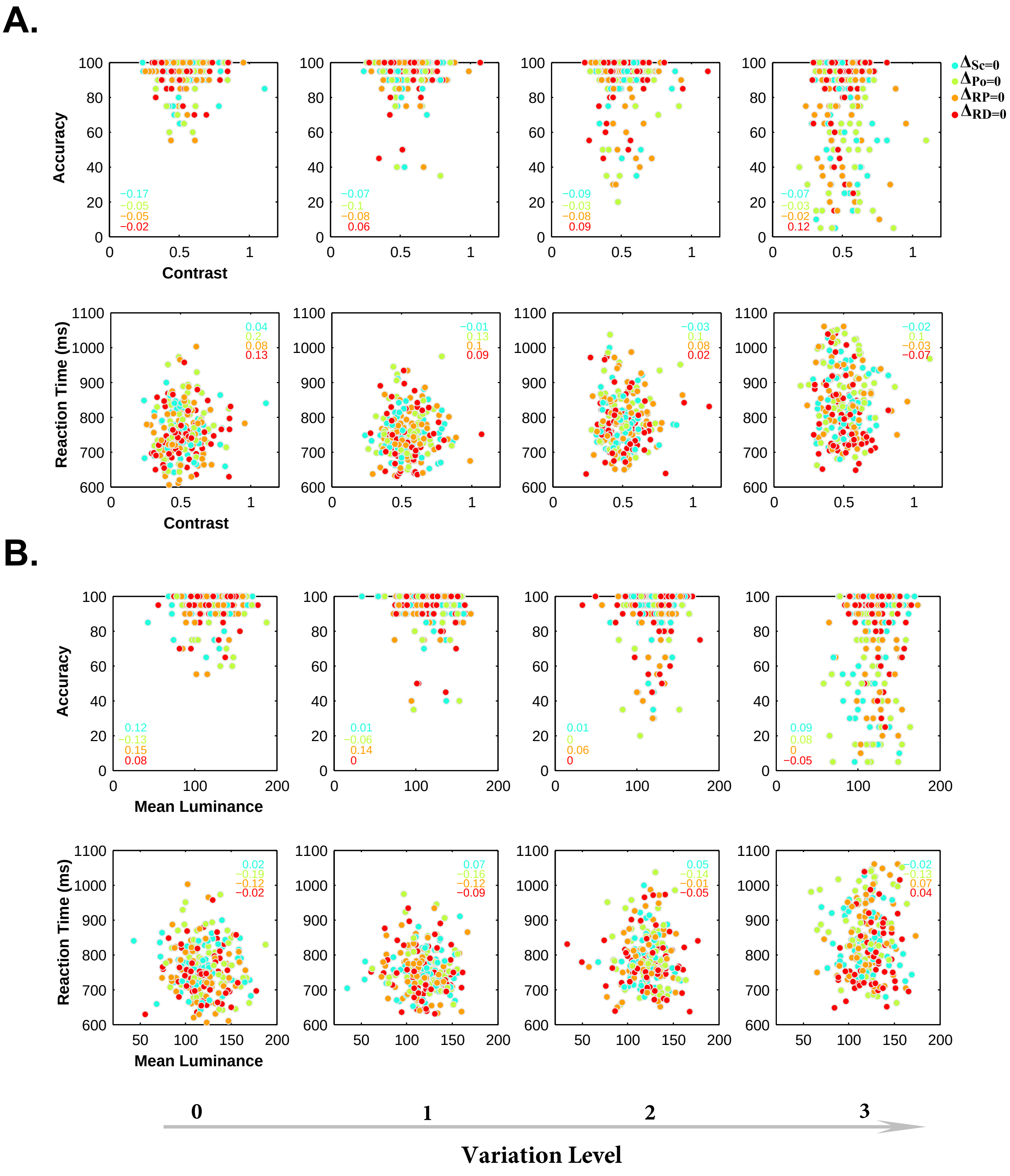

Figure S8. The correlation between low-level statistics (contrast and luminance) and the performance of human subjects, when objects had natural backgrounds. A. Correlation between the contrast of images (root mean square contrast) and human accuracy (top row) and reaction time (bottom row) for all levels and three variation conditions. Each point refers to an image and colors indicate an experimental condition. Correlation values are depicted in each scatter plot with corresponding colors (Pearson correlation). Significant correlations are specified using asterisks next to numbers. Scatter plots are plotted for all levels from level 0 (left) to level 1 (right). B. Correlation between the luminance of images (mean luminance of all pixels) and human accuracy (top row) and reaction time (bottom row) for all levels and three variation conditions.

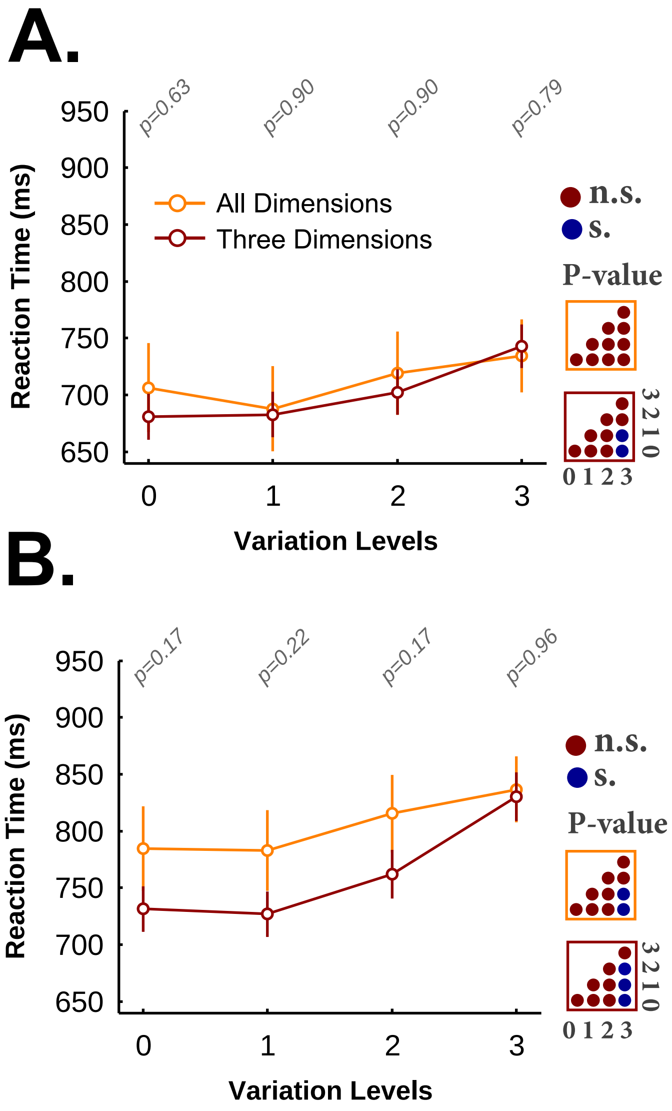

Figure S9. Average reaction time of human subjects in rapid invariant object categorization task. A. Average and standard error of the mean (SEM) of subjects' reaction time in the all- and three-dimension conditions, when objects had uniform background. The orange curve shows the reaction time when objects varied in all dimensions and the brown curve demonstrates the data for three dimensions. P values, depicted on the top, show whether the reaction time difference between the all- and three-dimension experiments are significant (Wilcoxon rank sum test). Color-coded matrices, on the right, show all possible pair-wise comparisons across levels, indicating whether or not the reaction time changes are statistically significant (Wilcoxon rank sum test; each matrix corresponds to one curve; see color of the frame). B. Average and SEM of subjects' reaction time in the all- and three-dimension conditions, when objects had natural backgrounds.

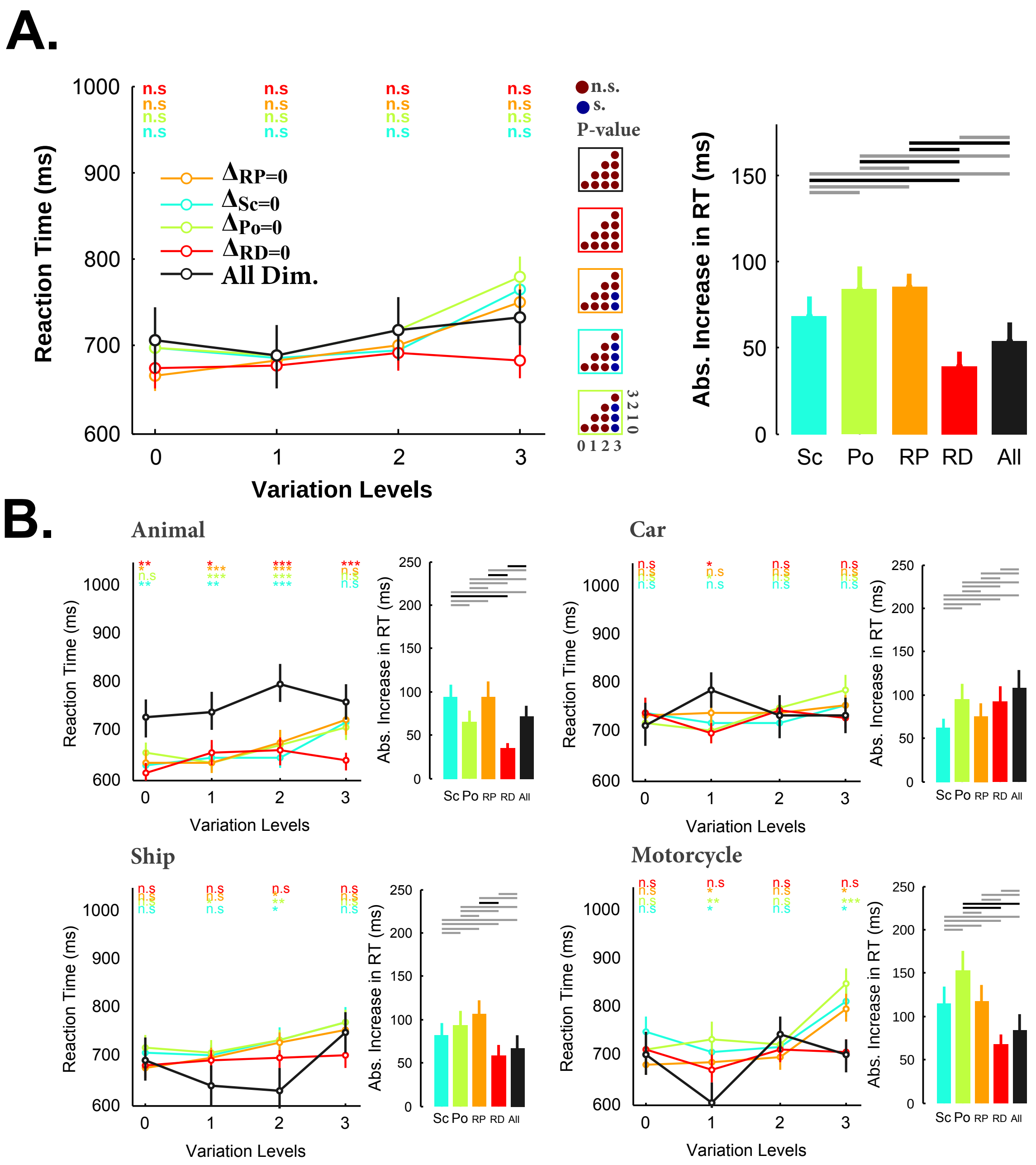

Figure S10. Average reaction time of human subjects in rapid invariant object categorization task for the all-dimension and different three-dimension conditions, when objects had uniform backgrounds. A. Left, average and standard error of the mean of subjects' reaction time in the all-dimension and different three-dimension conditions, when objects had uniform backgrounds. Each color refers to a condition. P values, depicted on the top, show whether the reaction time difference between the all-dimension and the other three-dimension conditions are statistically significant (Wilcoxon rank sum test). Color-coded matrices, on the right, show all possible pair-wise comparisons across levels, indicating whether the reaction time changes in each condition are statistically significant (Wilcoxon rank sum test; each matrix corresponds to one curve; see color of the frame). Right, absolute increase in reaction time between level 0 and level 3 (mean+/-STD). The horizontal lines on the top show whether the differences are significant (gray line: insignificant, black line: significant). B. Reaction time and absolute increase in reaction time for different conditions and object categories.

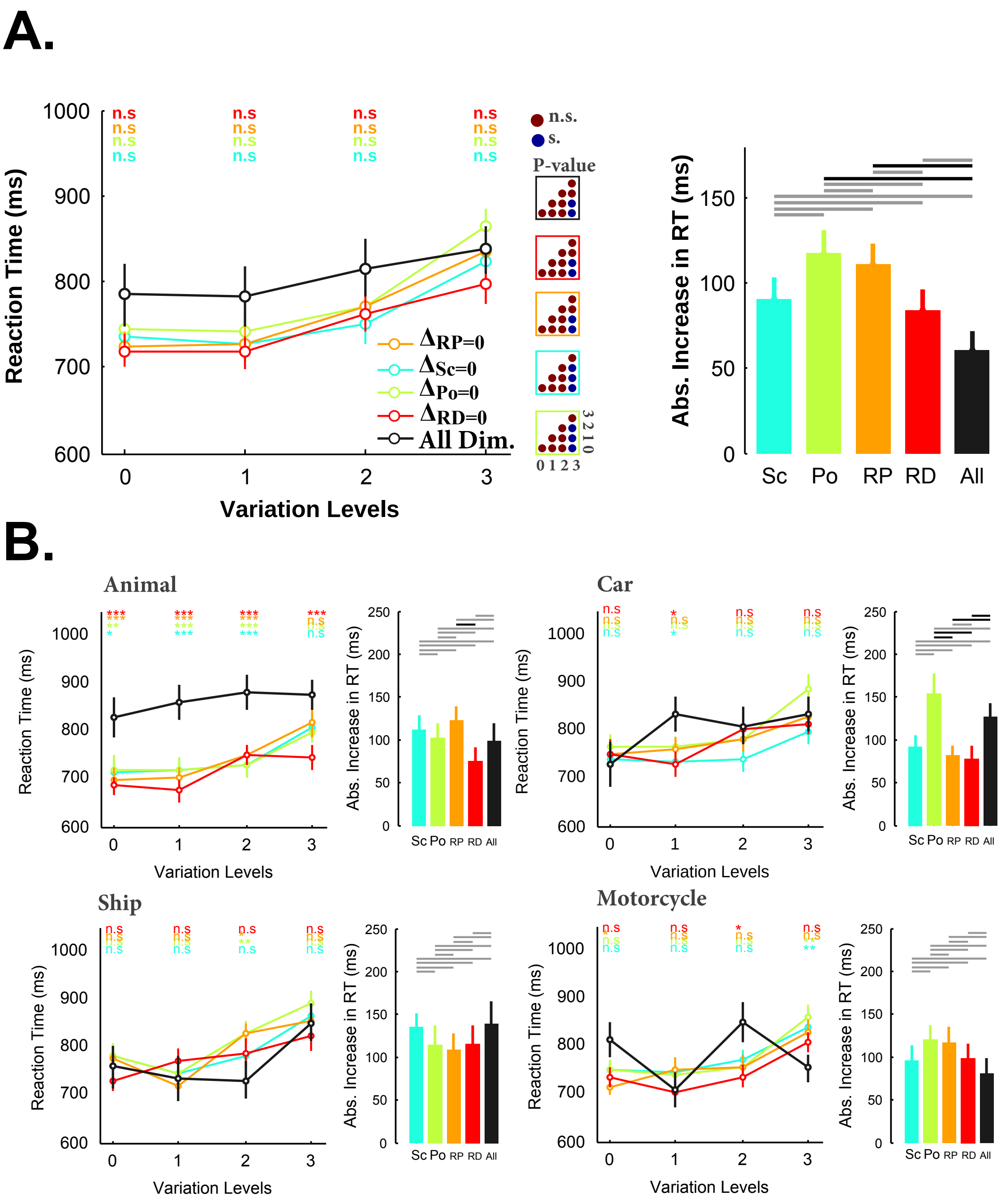

Figure S11. Average reaction time of human subjects in rapid invariant object categorization task for the all-dimension and different three-dimension conditions, when objects had natural backgrounds. A. Left, average and standard error of the mean of subjects' reaction time in the all-dimension and different three-dimension conditions, when objects had natural backgrounds. Each color refers to a three-dimension condition (p values and matrices were calculated using a similar approach to fig S10). Right, absolute increase in reaction time between level 0 and level 3 (mean+/-STD). The horizontal lines on the top show whether the differences are significant (gray line: insignificant, black line: significant). B. Reaction time and absolute increase in reaction time for different conditions and object categories.

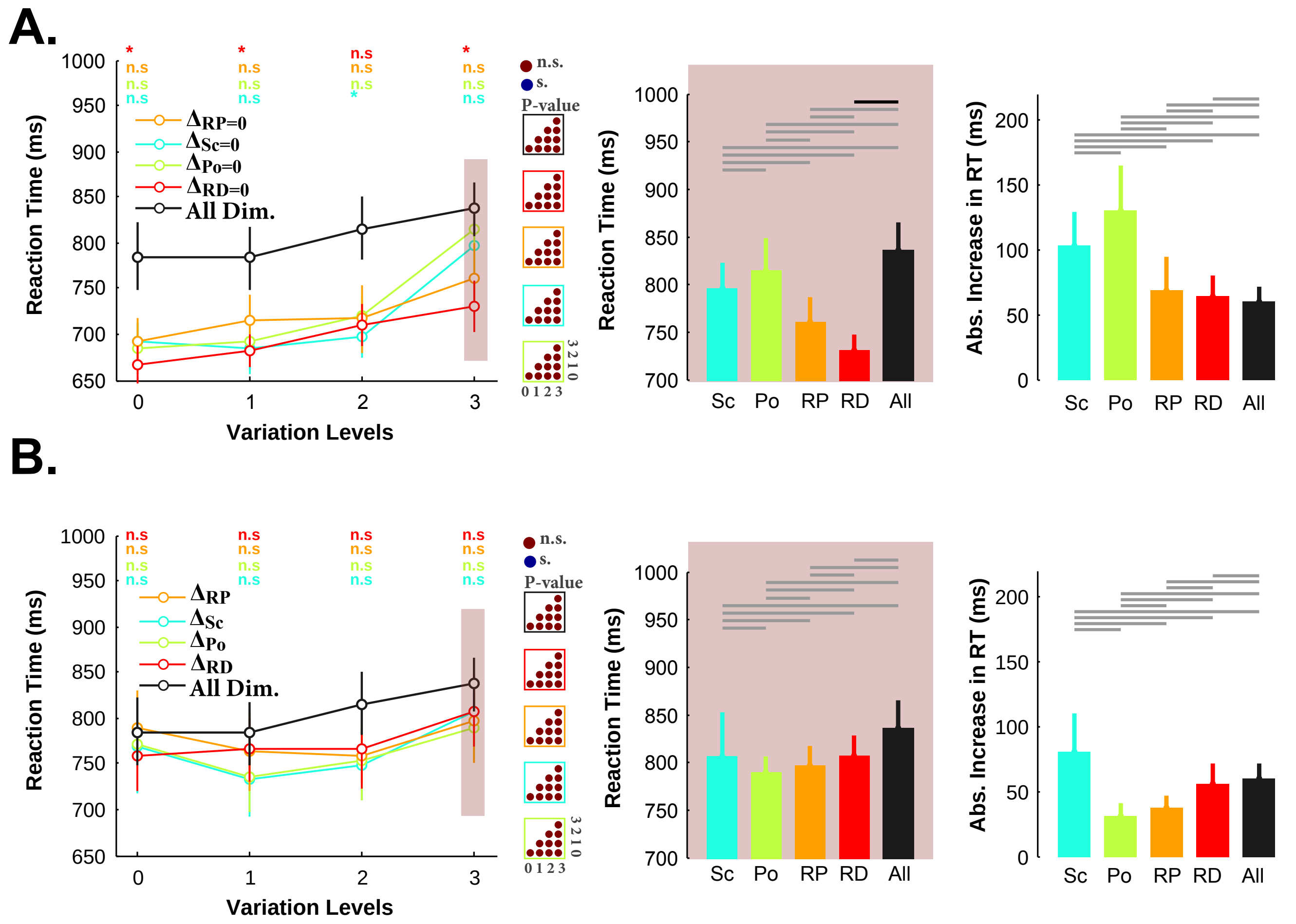

Figure S12. Reaction time of human subjects in ultra-rapid invariant object categorization task for the different three-dimension and one-dimension conditions, when objects had natural backgrounds. A. Left, average and standard error of the mean of subjects' reaction time in different three-dimension conditions. Each curve corresponds to one condition: $\Delta_{Sc} = 0$, $\Delta_{Po} = 0$, $\Delta_{RP} = 0$, $\Delta_{RD} = 0$ (as specified with different colors). Horizontal axis shows variation levels from level 0-3. Error bars are the standard deviation (STD). Color-coded matrices, on the right, show all possible pair-wise comparisons across levels, indicating whether the reaction time changes in each condition are statistically significant (Wilcoxon rank sum test; each matrix corresponds to one curve; see color of the frame). Middle, reaction times at the most difficult level for different three variation conditions (each bar corresponds to one condition). The horizontal lines on the top shows whether the differences are significant (gray line: insignificant, black line: significant). Right, absolute increase in reaction time between level 0 and level 3 (mean+/-STD). B. Left, average and standard error of the mean of subjects' reaction time in different one-dimension conditions (details of the plot are similar to A). Middle, reaction times at the most difficult level for different three variation conditions (each bar corresponds to a condition). Right, absolute increase in reaction time between level 0 and level 3 (mean+/-STD).
